# Cross-Disease Breathomics by PTR-TOF-MS: Multiclass Machine Learning and Network Remodeling Across Asthma, COPD, Cystic Fibrosis, and Lymphangioleiomyomatosis

**DOI:** 10.3390/ijms27083483

**Published:** 2026-04-13

**Authors:** Malika Mustafina, Artemiy Silantyev, Aleksandr Suvorov, Stanislav Krasovskiy, Marina Makarova, Alexander Chernyak, Olga Suvorova, Anna Shmidt, Daria Gognieva, Aleksandra Bykova, Nana Gogiberidze, Andrei Akselrod, Andrey Belevskiy, Sergey Avdeev, Vladimir Betelin, Abram Syrkin, Philipp Kopylov

**Affiliations:** 1 Department of Cardiology, Functional and Ultrasound Diagnostics, I.M. Sechenov First Moscow State Medical University (Sechenov University), 119048 Moscow, Russianana10.11@mail.ru (N.G.); syrkin_a_l@staff.sechenov.ru (A.S.);; 2 Pulmonology Research Institute under the Federal Medical and Biological Agency of Russia, 115682 Moscow, Russiamma123@list.ru (M.M.);; 3 Research Institute for Systemic Analysis of the Russian Academy of Sciences, 117218 Moscow, Russia; 4 World-Class Research Center “Digital Biodesign and Personalized Healthcare”, I.M. Sechenov First Moscow State Medical University (Sechenov University), 119048 Moscow, Russia; 5 Federal State Autonomous Educational Institution of Higher Education, N.I. Pirogov Russian National Research Medical University, 1 Ostrovityanova str., Bldg. 6, 117513 Moscow, Russia; 6 Pulmonology Department, I.M. Sechenov First Moscow State Medical University (Sechenov University), 119048 Moscow, Russia; olga.a.suvorova@mail.ru (O.S.);

**Keywords:** PTR-TOF-MS, breathomics, VOC, bronchial asthma, COPD, cystic fibrosis, lymphangioleiomyomatosis, machine learning, XGBoost, network analysis

## Abstract

Chronic obstructive and inflammatory lung diseases share overlapping clinical manifestations and spirometric features, complicating differential diagnosis and monitoring. In this study, we performed an integrative real-time proton-transfer-reaction time-of-flight mass spectrometry (PTR-TOF-MS) breathomics analysis to assess whether exhaled volatile organic compound (VOC) profiles enable multiclass discrimination among bronchial asthma (BA), chronic obstructive pulmonary disease (COPD), cystic fibrosis (CF), and lymphangioleiomyomatosis (LAM), with healthy individuals as controls. Breath VOC data from 843 subjects were analyzed using a stratified 70/30 train/test split. An ensemble feature selection strategy based on gradient boosting (XGBoost with SMOTE within cross-validation) identified stable VOC panels (top 25% selection probability), yielding 29 VOCs and 31 features including clinical covariates. On the independent test set, the VOC-only model achieved a macro-averaged one-vs-one (OvO) AUC of 0.866 (95% CI 0.829–0.903), while the combined model improved to 0.888 (95% CI 0.853–0.919), indicating modest value of clinical variables. Pairwise analysis demonstrated highest discrimination for CF (AUC up to 0.988), whereas BA and LAM showed lower sensitivity (<0.60), likely reflecting heterogeneity and limited sample size. Given differences in age, sex, BMI, and smoking status across cohorts, confounding effects were assessed, confirming that VOC signatures retain independent diagnostic information. Disease-specific VOC interaction networks revealed distinct remodeling patterns, with central metabolites not captured by univariate analysis. Overall, PTR-TOF-MS breathomics demonstrates proof-of-concept multiclass discrimination across chronic lung diseases.

## 1. Introduction

Obstructive and inflammatory lung diseases remain major causes of morbidity and premature mortality and are characterized by pronounced clinical and molecular heterogeneity. Bronchial asthma (BA) encompasses multiple endotypes with variable airway inflammation, remodeling, and response to therapy; accordingly, contemporary management explicitly emphasizes phenotype-directed assessment and treatment, including the integration of biomarkers of type 2 inflammation where appropriate [1]. Chronic obstructive pulmonary disease (COPD) is likewise heterogeneous, reflecting complex interactions among cigarette smoke or other exposures, airway and alveolar injury, systemic effects, and comorbidities, as summarized in current GOLD strategy documents [2]. Cystic fibrosis (CF) is a multisystem genetic disorder in which chronic airway infection and inflammation drive progressive structural lung damage despite transformative CFTR modulator therapies, and multidisciplinary surveillance remains essential in the modern era [3]. Finally, lymphangioleiomyomatosis (LAM) is a rare cystic lung disease affecting predominantly women and linked to dysregulated mechanistic target of rapamycin (mTOR) signaling, where clinical guidelines emphasize the roles of imaging, VEGF-D testing, and individualized therapy such as sirolimus [4].

Across these disorders, overlapping symptoms (dyspnea, cough, wheeze), shared physiologic readouts (airflow limitation), and treatment effects can blur clinical boundaries, especially in early disease or in the presence of comorbidities. In addition, standard lung function tests may be challenging in some patients (e.g., frail or severely dyspneic individuals), motivating complementary noninvasive approaches. This creates a strong rationale for complementary, noninvasive approaches capable of sampling airway biochemistry repeatedly and with minimal patient burden. Additional modern diagnostic methods, in combination with traditional ones, can improve diagnostic accuracy and support clinical decision-making.

Breathomics is the comprehensive profiling of volatile organic compounds (VOCs) in exhaled breath and an attractive candidate modality because exhaled air is readily accessible and can reflect both airway and systemic biochemistry without blood draws or sputum induction [5]. VOCs arise from endogenous metabolic pathways (including lipid peroxidation products generated under oxidative stress, reactive carbonyl chemistry, and downstream detoxification), from inflammatory cell activity, and from microbial metabolism in the airways [6]. At the same time, breath VOCs are susceptible to confounding by environmental exposures, diet, medications, and sampling methodology; therefore, rigorous standardization of collection, processing, and reporting is critical for clinical translation, as emphasized by European Respiratory Society technical standards and translational reviews on VOC biomarker development [7].

From an analytical perspective, gas chromatography–mass spectrometry (GC-MS) enables high-confidence compound identification, whereas proton-transfer-reaction mass spectrometry (PTR-MS) and its time-of-flight implementation (PTR-TOF-MS) provide high sensitivity and sub-second time resolution suitable for online breath analysis and real-time monitoring [8]. PTR-TOF-MS can acquire complete mass spectra within a fraction of a second and, owing to high mass resolving power, can separate isobaric ions and improve formula-level assignment of exact mass-to-charge (m/z) features [9]. In clinical environments, high temporal resolution is particularly valuable because it allows breath-resolved analysis across expiratory phases and supports continuous monitoring while simultaneously tracking room-air backgrounds that may influence apparent exhaled concentrations. Currently, research into the use of this method in the diagnosis of oncological, cardiovascular and respiratory diseases is becoming increasingly widespread [10,11,12].

A major remaining gap for clinical breathomics is the transition from binary case–control discrimination to multi-disease decision settings that mirror real-world differential diagnosis. Many VOC signals are not disease-specific but reflect shared axes such as oxidative stress and inflammation; therefore, multiclass modeling and cross-disease benchmarking are essential for identifying signatures that remain robust when several clinically relevant alternatives are considered simultaneously. Gradient-boosted decision trees (e.g., XGBoost) are well suited to this setting because they can capture non-linear feature interactions, handle mixed feature distributions, and provide ranked feature importance that can be aggregated across resampling for stability [13].

In parallel, disease biology is not only encoded in individual VOC intensities but also in co-variation structure: inflammatory, microbial, and host-metabolic pathways can rewire correlations among metabolites. Quantifying such network-level remodeling can therefore add a complementary layer of information and help prioritize VOCs that occupy central positions in disease-specific interaction graphs. Distance correlation is a flexible dependence measure that can detect both linear and non-linear associations and is therefore suitable for constructing weighted interaction graphs in heterogeneous biomedical datasets [14].

In the present study, we integrated PTR-TOF-MS breath datasets previously acquired in cohorts with BA, COPD, CF, and LAM, alongside healthy controls [15,16,17,18]. This integrated design enables cross-disease benchmarking in a unified analytical framework and comparative network analysis across distinct disease mechanisms (e.g., type 2 inflammation in subsets of BA, smoking-related oxidative injury and systemic effects in COPD, chronic infection-driven airway inflammation in CF, and mTOR-driven cystic lung remodeling in LAM). We aimed to develop and interpret multiclass breathomics models that operate in a clinically realistic setting and to relate model-discriminative VOC panels to network-level remodeling across diseases.

## 2. Results

### 2.1. Dataset Composition

One breath sample was obtained from each subject. The integrated dataset included 441 breath measurements across patients (each contributing one breath sample) (221 males and 220 females) and 402 breath measurements of healthy controls (154 males and 248 females). Among patient measurements 160 (36%) corresponded to BA, 128 (29%) to COPD, 102 (23%) to CF and 51 female patients (12%) to LAM. The age of all patients was from 18 to 89 years; however, the CF cohort was the youngest. Furthermore, patients with CF had the lowest BMI compared with the control group and other patients (*p* = 0.02). COPD patients had a higher prevalence of current/former smoking (*p* < 0.01), while all patients with CF were nonsmokers. All patients had obstructive abnormalities on pulmonary function testing, but the greatest small airway involvement was observed in patients with CF (*p* < 0.01). Table 1 summarizes the baseline characteristics of the study population. However, detailed characteristics of all cohorts of patients are presented in our previous studies [15,16,17,18]. 

The study included 843 subjects distributed across five diagnostic groups. The entire cohort was randomly partitioned into a training subset (70%, *n* = 590) and a validation subset (30%, *n* = 253) using stratified sampling. Class proportions were preserved across all subsets to ensure a valid evaluation of diagnostic models (Table S1). The control group was the largest, comprising 47.6% of the total dataset. Among the pathological conditions, bronchial asthma (BA) represented the highest proportion (19.0%), whereas lymphangioleiomyomatosis (LAM) accounted for the smallest share (6.0%), reflecting either the real-world prevalence of these conditions or the specifics of cohort recruitment. Class balance between the training and validation subsets was maintained with precision to within tenths of a percent, confirming the adequacy of the stratification procedure.

**Figure 1:**
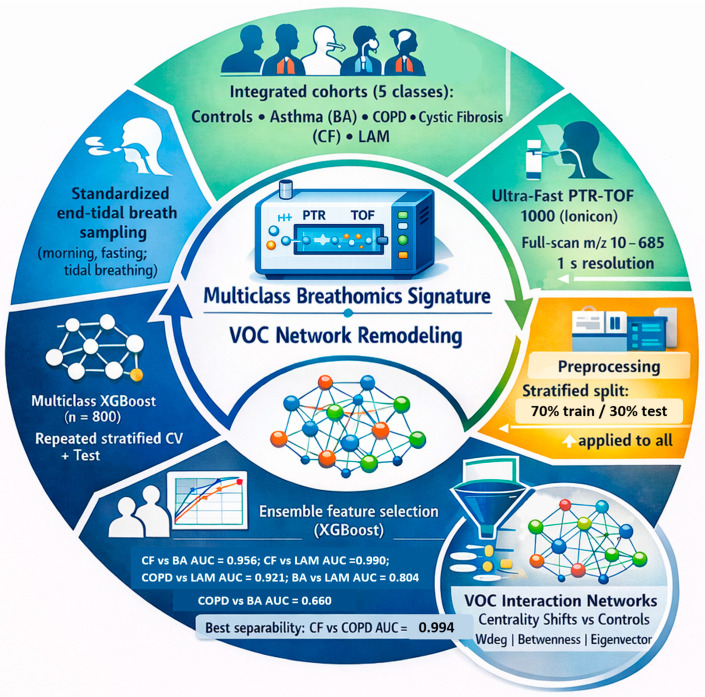
Circular workflow (Cohorts, breath sampling, PTR-TOF-MS acquisition, preprocessing, feature selection, multiclass XGBoost).

Table 1 summarizes pooled baseline characteristics; detailed cohort-specific characteristics have been reported previously [15,16,17,18]. The circular workflow (cohorts, breath sampling, PTR-TOF-MS acquisition, preprocessing, feature selection, multiclass XGBoost) is presented in Figure 1.

### 2.2. Multiclass Classification Performance and Assessment of Confounding Effects

To reduce dimensionality and identify the most diagnostically significant VOCs, an ensemble feature selection method based on gradient boosting (XGBoost version 3.2.0) was implemented. All preprocessing steps were implemented without information leakage: scaling/normalization was fitted on training data only and applied to the corresponding validation/test data, and minority-class oversampling (SMOTE) was performed strictly within each cross-validation training fold as part of an imbalanced-learn pipeline (scaler → SMOTE → XGBoost). The procedure included the following steps: stratified resampling, class balancing using SMOTE, feature importance assessment, aggregation and selection.

Based on the probability distribution of feature selection, two feature panels were constructed: (1) a comprehensive panel incorporating clinical covariates, and (2) a panel based exclusively on volatile organic compounds (VOCs). For each panel, gradient boosting classification models (XGBoost) were trained, with regularization hyperparameters optimized using successive halving random search (HalvingRandomSearchCV). Model performance was evaluated on an independent test set by computing the macro-averaged area under the ROC curve (one-vs-one, OvO), pairwise metrics, and binary performance indicators (sensitivity, specificity, and predictive values), with 95% confidence intervals estimated via nonparametric bootstrapping (500 iterations).

The results of the stability selection procedure are presented as a table of selection probabilities for each of the 123 initial features (Table S2). The threshold for inclusion in the final feature panel was set at the 75th percentile of the selection probability distribution among features with non-zero stability.

Participants from all five clinical groups (BA, COPD, CF, LAM, and controls) were recruited and measured at a single site within the same overall study period (January 2023—December 2024) using the same PTR-TOF-MS platform, and harmonized sampling workflow. This design minimizes classical multi-center or inter-study batch effects and supports direct comparison of VOC profiles across groups. However, baseline characteristics differed substantially between groups, particularly age, sex distribution (LAM female-only), smoking status (higher prevalence in COPD), and BMI (lower in CF) (Table 1), all of which may influence exhaled VOC signatures.

**Figure 2:**
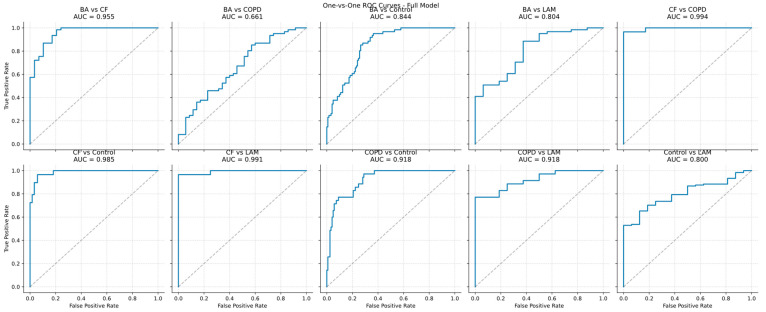
Multiclass ROC analysis of O-v-O for the models VOCs with covariates (The Precision–Recall curves for these models are shown in Figure S1A). Solid blue lines represent ROC curves for pairwise class comparisons, while gray dashed lines indicate the reference line (random classifier). AUC: Area Under the Curve; BA: bronchial asthma; COPD: chronic obstructive pulmonary disease; CF: cystic fibrosis; LAM: lymphangioleiomyomatosis; ROC: Receiver Operating Characteristic.

**Figure 3:**
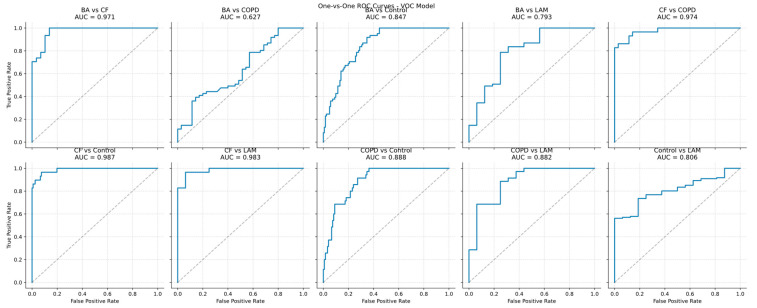
Multiclass ROC analysis of O-v-O for the VOC-only models (The Precision–Recall curves for these models are shown in Figure S1B). Solid blue lines represent ROC curves for pairwise class comparisons, while gray dashed lines indicate the reference line (random classifier). AUC: Area Under the Curve; BA: bronchial asthma; COPD: chronic obstructive pulmonary disease; CF: cystic fibrosis; LAM: lymphangioleiomyomatosis; ROC: Receiver Operating Characteristic.

To quantify the potential impact of these covariates, we trained a multiclass baseline model using clinical variables (age, gender, BMI, and smoking status) plus VOC-features under the same 70/30 train/test split and evaluation scheme as the VOC-only based models. We then compared covariate-only performance with VOC-only performance (based on 75th percentile threshold) and with a combined model including both VOC features and covariates, thereby estimating the incremental predictive value of breathomics beyond standard clinical descriptors (Figure 2 and Figure 3).

Clinical covariates (age, sex, body mass index (BMI), and smoking status) were incorporated into the analysis as protected variables, retained in all models to control for potential confounding. In the final comprehensive panel comprising 31 features, the covariates age and BMI were selected with stability probabilities ≥0.75, whereas sex and smoking status did not reach the selection threshold (probability = 0.000). These findings indicate that, within this dataset, age and BMI exhibit more robust associations with the diagnostic groups than sex and smoking status, after accounting for VOC spectral features.

Based on the 75th percentile threshold, two feature panels were constructed for model training:

Full panel (VOCs + Covariates), *n* = 31: 83.08564, 95.082, 95.05365, 97.09225, 96.05719, 159.0712, 97.10352, Age, 235.21074, 71.05515, 71.08162, 181.01353, 149.10438, 85.09638, 85.0697, 105.93981, BMI, 73.06527, 107.9565, 79.05395, 109.07056, 55.03962, 109.09555, 119.95525, 103.07641, 118.0709, 58.94678, 45.99156, 329.83984, 77.05868, 47.04044.

VOC-only panel (VOCs only), *n* = 29: 83.08564, 95.082, 95.05365, 97.09225, 96.05719, 159.0712, 97.10352, 235.21074, 71.05515, 71.08162, 181.01353, 149.10438, 85.09638, 85.0697, 105.93981, 73.06527, 107.9565, 79.05395, 109.07056, 55.03962, 109.09555, 119.95525, 103.07641, 118.0709, 58.94678, 45.99156, 329.83984, 77.05868, 47.04044.

The error matrices for the training and test samples are presented in Table S3A,B.

On the test set, the full model (VOCs + covariates) achieved a macro-averaged OvO AUC of 0.888 (95% CI 0.853–0.919), compared with 0.866 (95% CI 0.829–0.903) for the VOC-only model, indicating a modest positive contribution of clinical covariates. The highest discriminative performance was observed for cystic fibrosis (CF): the pairwise AUC for CF versus controls reached 0.986 (95% CI 0.966–0.997) in the full model and 0.988 (95% CI 0.969–0.999) in the VOC-only model. Sensitivity for CF was also high (0.722 and 0.759, respectively), demonstrating robust identification of this class. In contrast, bronchial asthma and lymphangioleiomyomatosis (LAM) were the most challenging to classify, with test sensitivities not exceeding 0.60. The wide confidence intervals for LAM metrics (e.g., NPV 0.213 [0.113–0.313]) reflect substantial uncertainty due to the small sample size.

The primary model performance metrics, including the macro-averaged area under the ROC curve in the one-vs-one (OvO Macro AUC) framework and pairwise binary performance measures with 95% confidence intervals (CIs), are presented in Table S4. All binary metrics were derived using thresholds optimized according to Youden’s index on the training set, with 95% confidence intervals estimated via bootstrapping (500 replicates).

The full model (including age, sex, BMI, and smoking status) achieved higher AUC values for most class pairs, particularly those involving COPD—for example, COPD vs. Control (0.918 vs. 0.888) and COPD vs. LAM (0.921 vs. 0.889)—as well as BA vs. COPD (0.660 vs. 0.629), consistent with known associations of COPD with age and smoking. The VOC-only model, however, showed comparable or higher sensitivity for BA vs. CF (0.610 vs. 0.528) and CF vs. Control (0.759 vs. 0.722), indicating independent diagnostic value of VOC profiles. Both models demonstrated high specificity (>0.9) across most comparisons, especially those involving CF or COPD. Low NPVs in LAM-related pairs (e.g., Control vs. LAM, NPV 0.213) reflect the small sample size of this class and corresponding statistical uncertainty. Overall, macro-averaged AUCs confirm a slight advantage of the full model (0.888 vs. 0.866), although VOC features alone retain strong discriminative performance.

### 2.3. Comparative Characteristics of VOCs Between Groups

Between-group analysis was performed in five study groups (BA, COPD, LAM, CF and healthy volunteers) for each of the 30 selected VOCs (top 25% importance in the gradient boosting (XGBoost) model) (*p* < 0.001); post hoc Mann–Whitney tests (Holm–Bonferroni corrected) revealed disease-specific patterns. Comparative characteristics of the presence of VOCs in various chronic lung diseases are shown in Table 2.

When analyzed separately by patient groups, the lowest values of abundance of phenol (m/z 95.05, median abundance 0.019) and indole (m/z 118.07, median abundance 0.003) ions were found in patients with CF compared to control groups, patients with COPD, BA, and LAM (*p* < 0.001). This may indicate a potential role for these VOCs as markers of deficiency in CF. In contrast, patients with COPD showed the greatest increase in the abundance of ions with m/z 71.06, 73.07, 79.05 and 97.09 (median abundance 0.36, 0.17, 0.04 and 0.04, respectively) compared to the control group, patients with bronchial asthma, CF and LAM (*p* < 0.001). BA was characterized by a moderate increase in the number of VOCs: m/z 95.05 (putative, phenol), median abundance 0.02, m/z 118.07 (putative indole), median abundance 0.03 and m/z 149.10 (putative diethanolamine), median abundance 0.02, were higher than in the CF group (*p* < 0.001), but lower than in the LAM group (*p* < 0.001). The LAM group did not show a VOC with consistently higher abundance than all other groups under the current feature set and sample size. However, the following features were noted: VOC with m/z 118.07 (putative indole, median abundance 0.060) was significantly higher than in CF and COPD (*p* < 0.001), indistinguishable from BA; VOC with m/z 159.071 (putative thiazole, median abundance 0.112) was higher than in CF, COPD and control group (*p* < 0.001) and also indistinguishable from BA.

Thus, the most differentiated group was CF, which exhibited a systemic decrease in the intensity of key VOCs (phenol m/z 95.05 and indole m/z 118.07) compared to all other conditions; COPD was characterized by an increase in a number of VOCs, while LAM and BA had less pronounced, but statistically significant profiles that differed from CF and control group. All selected VOCs showed global significance in the Kruskal–Wallis test (*p* < 0.001), which supports their statistical association with disease classes in this dataset.

### 2.4. Network Remodeling of VOC Interactions

To identify disease-specific remodeling of associative structures among the selected VOCs, weighted graphs were constructed using signed distance correlation. Four centrality measures were computed: weighted degree, betweenness centrality, eigenvector centrality, and Katz centrality. In addition, maximal weighted clique analysis was performed using an edge inclusion threshold (r) corresponding to a moderate effect size (d = 0.5). All centrality metrics were normalized using Min–Max scaling within each class, and deviations were calculated relative to the control group. For each class and metric, deviations exceeding the 5th and 95th percentiles of the overall deviation distribution (adaptive thresholds) were classified as strong (*). The results are summarized in Supplementary Materials (Supplementary network_analysis_summary.txt).

**Figure 4:**
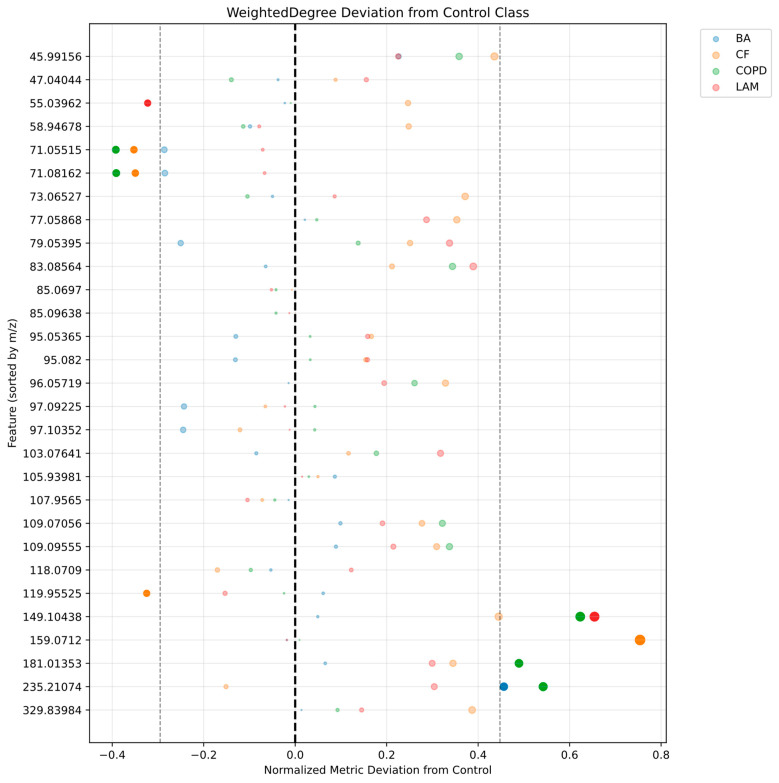
Weighted degree deviation from control by mass spectrometry features. The black dashed vertical line indicates zero deviation (i.e., no difference from the control group). BA: bronchial asthma; COPD: chronic obstructive pulmonary disease; CF: cystic fibrosis; LAM: lymphangioleiomyomatosis; m/z: mass to charge. The size of the node is determined by the total importance of the feature.

Weighted degree deviation from control group for selected VOC signals are presented in Figure 4.

In the control network, the highest weighted degree values were observed for nodes 85.07 and 85.10 (≈10.51), followed by nodes 97.10, 97.09, 71.08, and 71.06 (10.16–10.24), as well as nodes 119.96 (10.06) and 107.97 (10.05). These nodes also exhibited the highest eigenvector and Katz centrality values, confirming their dominant roles in the network structure of healthy individuals.

In the BA network, a strong positive deviation in weighted degree was observed for node 235.21 (Δ = +0.456), representing the only node exceeding the upper threshold (0.448). No strong negative deviations were detected; however, nodes 71.05515 and 71.08162 showed values close to the lower threshold (Δ ≈ −0.286). Eigenvector centrality analysis revealed a pronounced negative deviation for node 71.06 (Δ = −0.306), indicating a loss of its central role. No nodes exceeded the positive threshold, although nodes 235.21 and 45.99 approached it. Overall, the BA network is characterized by increased importance of node 235.21 alongside weakening of node 71.06.

In the COPD network, strong positive deviations in weighted degree were observed for nodes 235.21 (Δ = +0.542), 181.01353 (Δ = +0.489), and 149.10 (Δ = +0.623), while strong negative deviations were detected for nodes 71.06 and 71.08 (Δ ≈ −0.39). Eigenvector and Katz centrality analyses showed consistent patterns, with increased centrality for nodes 149.10 (Δ = +0.583), 235.21 (Δ = +0.482), 83.06 (Δ = +0.346), and 96.06 (Δ = +0.312). The same 71.xx nodes exhibited strong decreases. These findings indicate substantial network reorganization, with emergence of new central hubs while previously dominant nodes lose influence.

In the CF network, the strongest positive deviation in weighted degree was observed for node 159.07 (Δ = +0.754). Additional increases were found for node 96.06 (Δ = +0.329), while strong negative deviations were detected for nodes 71.06 and 71.08 (Δ ≈ –0.35) and node 119.96 (Δ = –0.325). Centrality analysis revealed marked increases for nodes 159.07 (Δ = +0.761), 96.06 (Δ = +0.451), and 149.10 (Δ = +0.471), whereas node 119.96 showed a strong decrease (Δ = –0.343). Nodes in the 71.xx range consistently exhibited reduced centrality. A distinctive feature of the CF network is the dominant role of node 159.07 and the emergence of phenolic nodes (95.05 and 95.08) within tightly connected subnetworks.

In the LAM network, node 149.10 showed a strong positive deviation in weighted degree (Δ = +0.654), while node 55.04 exhibited a significant negative deviation (Δ = –0.323). Eigenvector and Katz centrality analyses revealed increased importance for nodes 149.10 (Δ = +0.681) and 103.08 (Δ = +0.336). Notably, nodes in the 71.xx range did not show strong deviations (Δ ≈ −0.06), in contrast to other disease groups. The LAM network also displayed the highest level of integration, with the largest number of highly central nodes and the largest clique (24 nodes).

Analysis of betweenness centrality revealed disease-specific changes in network bridging roles. Strong positive deviations were observed for node 79.05 (Δ = +0.735) and node 159.07 (Δ = +0.370) in BA, node 47.04044 (Δ = +0.852) in COPD, node 235.21 (Δ = +0.926) in CF, and node 159.0712 (Δ = +0.370) in LAM. In contrast, strong negative deviations were consistently observed for node 181.01 across all disease groups (Δ = –0.412 to –1.000), indicating loss of its bridging role. Node 149.10 also showed reduced betweenness in all groups except BA. These results suggest that disease progression is associated with replacement of key network bridges by disease-specific nodes.

The exact centrality values are provided in Supplementary Table S7 (Supplementary network_analysis_summary.txt), and weighted correlation networks for all groups are shown in Figure S2.

Overall, centrality deviation profiles demonstrate disease-specific network remodeling relative to controls, identifying VOC nodes with the most pronounced shifts in network influence. Importantly, these patterns are not fully captured by univariate analysis, underscoring the added value of network-based approaches in breathomics.

## 3. Discussion

### 3.1. Principal Findings and Clinical Framing

In this study, we performed an integrated breathomics analysis using PTR-TOF-MS to evaluate whether exhaled VOC profiles enable multiclass discrimination across clinically overlapping chronic lung diseases (BA, COPD, CF, and LAM) within a unified analytical framework. Unlike traditional case–control designs, this approach reflects a more clinically relevant scenario in which several competing diagnoses must be distinguished simultaneously.

The main finding is that PTR-TOF-MS breathomics provides robust multiclass discrimination with compact and stable VOC feature panels, achieving macro-averaged test AUC values of 0.866 for VOC-only models and 0.888 when combined with clinical covariates. Importantly, the improvement with covariates was modest, indicating that VOC profiles retain substantial independent diagnostic value beyond standard clinical descriptors.

A key methodological advance of this work is the use of stability-based feature selection, which allowed the identification of reproducible VOC signals across resampling. The final feature panels (29 VOCs and 31 features including covariates) were derived using a stability threshold, ensuring that model performance is not driven by unstable or dataset-specific features. This supports the robustness of the proposed breathomics signatures in a multiclass setting.

Class-pair discrimination was strongest for CF, including CF vs. COPD (AUC = 0.994) and CF vs. control group (AUC = 0.985). When exhaled air was analyzed in patients with CF, a significant decrease in VOC concentrations was observed when compared with the control group (see Table 2). This may reflect to the influence of chronic respiratory bacterial infection as well as intestinal microflora in the context of pancreatic insufficiency, which is consistent with our previous results [18]. COPD was also well separated from the control group (AUC = 0.918) and from LAM (AUC = 0.918). The specific exhaled air profile in patients with COPD may be largely due to VOC exposure due to smoking and the development of neutrophilic inflammation. In contrast, class-wise OvO metrics showed lower sensitivity for BA and LAM (<0.60). Several factors likely contribute. BA is biologically heterogeneous (type 2-high vs. type 2-low, variable airway remodeling, variable corticosteroid exposure), and VOC profiles may therefore be less consistent across patients than in conditions dominated by chronic infection and neutrophilic inflammation. LAM is a rare disease with smaller cohort sizes and may require either more features, orthogonal biomarkers, or longitudinal context to improve recall.

Importantly, this study also explicitly addressed the role of potential confounding variables, including age, sex, BMI, and smoking status, which differed substantially across cohorts. While age and BMI showed stable contributions to model performance, VOC-only models remained highly informative, supporting the presence of disease-related biochemical signal beyond demographic differences. Nevertheless, the observed performance patterns should be interpreted with caution, as residual confounding cannot be fully excluded in this integrated dataset.

### 3.2. Biological Interpretation of the Most Significant VOCs

PTR-TOF-MS provides high-resolution, real-time VOC profiling, but compound identification remains putative when it is based primarily on exact m/z matching. Mechanistic interpretation should therefore be treated as hypothesis-generating unless confirmed by orthogonal methods such as targeted GC-MS with standards and retention time confirmation [19,20]. Based on the presumed annotation of some VOCs, we attempted to identify consistent biochemical pathways that explain the significance of their detection in exhaled air in patients with chronic respiratory diseases.

When analyzing deviations from the control of VOCs in exhaled air, pronounced deviations of VOCs with m/z 95.05 (annotated as phenol) and 118.07 (annotated as indole) are observed in CF and COPD. These data are consistent with our previously described results from the volatility of patients with CF, which is likely due to changes in the composition of the intestinal microflora due to long-term antibiotic therapy [17,18]. A similar effect is possible in patients with COPD who experience frequent exacerbations. In addition, elevated phenol concentrations in patients with COPD have been described previously [21,22]. The decrease in indole abundance in patients with COPD is also consistent with the data of Gaida A. et al. [23].

VOCs with m/z 83.09, which are presumed to be cyclohexenes, are of great importance in exhaled air analysis across all four disease classes (CF, BA, LAM and COPD). However, the greatest deviation from control was observed in patients with COPD (Figure 3). Researchers Pizzini A. et al. also found an increase in these biomarkers in patients with COPD [24]. The origin of these VOCs may be due to the pyrolysis of cycloalkanes at high smoking temperatures due to lipid peroxidation.

The VOC with m/z 235.21 (presumably terpene) was also found to be the most significant for CF and COPD. This biomarker may be a product of secondary oxidation of aromatic precursors, or an indicator that the airway environment actively “oxidizes” incoming VOCs due to chronic neutrophilic inflammation characteristic of both CF and COPD, and may also be a product of smoking in patients with COPD. Furthermore, chronic bacterial infection in both of these diseases can biotransform both aromatic precursors into aromatic aldehydes and monoterpenes into terpene derivatives. The VOC with m/z 71.06 (annotated as 2-pentanone), a significant nodule in COPD and CF, may be a component of the oxidative carbonyl profile (a result of lipid peroxidation of polyunsaturated fatty acids) with possible microbial contribution (microbial fermentation and catabolism of amino or fatty acids). The increase in 2-pentanone is consistent with previously described results from exhaled breath analysis in COPD patients using an ion mobility spectrometry with gas chromatographic pre-separation [25]. A biomarker with m/z 159.07 (annotated as thiazole or thiazolium derivatives) demonstrated the highest value in CF. Studies have shown that sulfur- and nitrogen-containing volatile organic compounds (VOCs) are often the byproducts of chronic respiratory infections such as *Pseudomonas aeruginosa* [26,27].

As a result, the most significant deviation from control was the change in exhaled air profiles in patients with CF (characterized by pronounced long-term bacterial colonization of the lower respiratory tract) and in patients with COPD (those with the status of active smokers or a history of smoking and the resulting exposure to external VOCs and lipid peroxidation products). Thus, it is possible to formulate a hypothesis about three main sources of VOCs that played key roles in the differences in the spectra of exhaled air measured by proton mass spectrometry. The first source is oxidative stress and lipid peroxidation. Reactive oxygen and nitrogen species generated as a result of cellular inflammation and epithelial damage can induce peroxidation of polyunsaturated fatty acids, leading to the formation of a spectrum of aldehydes, alkenes, and oxygen-containing moieties that are detectable as VOC signals in exhaled air. This pathway is expected to play an important role in smoking-related COPD and CF, where chronic neutrophilic inflammation and persistent infection can maintain oxidative stress. The second source is microbial and intercellular metabolism. In CF, chronic colonization and frequent antibiotic exposure may alter microbial pathways, volatile metabolite production, and host detoxification responses; these mechanisms likely contribute to the clear distinction between CF and other classes in the presented results. The third possible source is of external origin: smoking products in patients with COPD or components of inhalation therapy [28].

LAM represents a distinct mechanistic category, linked to dysregulated mTOR signaling and cystic lung remodeling, with guideline emphasis on imaging features and biomarkers such as VEGF-D [4]. Breathomics may capture downstream metabolic consequences of remodeling and altered cellular signaling, but the signal may be subtler and more sensitive to confounding. This provides a plausible biological rationale for the lower LAM sensitivity observed and supports future designs that combine VOCs with clinical and laboratory variables to improve detection of rare disease classes. In general, patients with bronchial asthma and LAM, who had smaller clinically significant functional differences from the control group, in contrast to COPD and CF, also demonstrated less pronounced deviations in exhaled air profiles when compared with the control group (Figure 3).

### 3.3. Network Remodeling and Centrality as Complementary Signal

A distinctive aspect of this work is the focus on VOC interaction structure rather than only marginal feature shifts. Breath VOCs can share biochemical precursors, arise from linked redox pathways, reflect common microbial processes, or respond to shared exogenous exposures. Capturing such dependencies may improve interpretation and can help prioritize VOCs that are influential within disease-specific interaction patterns.

Using distance correlation—a dependence measure capable of detecting both linear and non-linear associations [14]—we constructed weighted interaction graphs and quantified node centrality metrics (weighted degree, weighted betweenness, eigenvector centrality and Katz centrality). Centrality deviations relative to controls revealed disease-specific patterns, supporting the idea that disease processes not only shift VOC levels but also rewire the structure of VOC co-variation. For example, the results highlight nodes with consistent multi-dimensional deviations across diseases as well as nodes with class-selective changes in bridging-related properties. Such bridging nodes are conceptually interesting because they may connect distinct biochemical modules, and they are pragmatic targets for targeted identification and validation: a metabolite that repeatedly changes its network position across disease states may encode higher-level pathway perturbations that are not evident from intensity differences alone. In our study, the key such nodes were VOCs with m/z 97.09, 95.05, 71.06, 235.21, 149.10, 159.07, 96.06.

Overall, the network perspective complements multiclass classification by offering mechanistic hypotheses and an additional prioritization layer for biomarker refinement. In future studies, coupling centrality shifts to pathway-level annotation (after targeted identification) may help interpret whether a VOC reflects lipid peroxidation, microbial activity, xenobiotic exposure, or host detoxification in a disease-specific manner.

### 3.4. Methodological Considerations and Limitations

Several methodological considerations should be acknowledged. First, the integrated dataset pools cohorts originally acquired for disease-specific analyses [15,16,17,18]. Even with a common analytical platform and standardized sampling principles, between-cohort differences in recruitment, seasonality, fasting state, and medication patterns may introduce residual confounding. Both ERS technical guidance and translational reviews emphasize the need to document and control these variables and to track environmental background VOCs that can influence apparent exhaled concentrations [7]. Our results may partly reflect systematic confounding, because CF participants were younger, with lower BMI, and uniformly non-smokers, whereas COPD participants were older and more frequently current or former smokers (Table 1). Since these factors are known to influence breath VOC composition, residual confounding cannot be fully excluded despite explicit modeling. Comparison of covariate-only, VOC-only, and combined models indicates that VOC profiles provide substantial independent discriminatory signal; however, clinical variables (especially age and BMI) contribute additional information and may partly shape class separation.

Second, class imbalance and limited sample size in underrepresented groups, particularly LAM, may affect model stability and reduce sensitivity in the multiclass setting. Although SMOTE-based resampling was applied within cross-validation folds to mitigate imbalance, synthetic data generation introduces additional assumptions and may not fully capture the biological variability of rare diseases. This is consistent with the observed lower sensitivity for BA and LAM compared to CF and COPD, as well as wider confidence intervals in performance metrics [29].

Third, VOC annotation remains putative in the absence of confirmatory analytical chemistry. Exact-mass assignment can be ambiguous because multiple compounds and fragments may share similar m/z, and adduct formation or humidity-related ion chemistry can complicate interpretation [30]. Finally, this work is cross-sectional. Breath VOCs are dynamic and can change with exacerbations, infections, environmental exposures, and treatment responses. Longitudinal sampling may be particularly important for BA (episodic activity) and for LAM (gradual progression and therapy effects). External validation in independent, prospectively recruited multiclass cohorts remains essential before clinical deployment [7].

Despite the described limitations, the results obtained and the network approach introduced here can be extended to longitudinal network dynamics, enabling tests of whether treatment shifts VOC interaction structure toward control-like patterns and whether early network changes predict clinical outcomes. These steps are required to move breathomics from promising discovery studies toward validated, implementable tools in respiratory medicine.

## 4. Materials and Methods

### 4.1. Study Cohorts and Ethical Approval

The integrated dataset pooled breathomics data from previously published disease-specific cohorts (BA, COPD, CF, and LAM) with corresponding controls. All participants were recruited prospectively at a single clinical site between January 2023 and December 2024 and measured using the same PTR-TOF-MS (Ionicon, Innsbruck, Austria) platform under a harmonized protocol. Breath sampling was performed in the morning under fasting conditions during clinical stability (no exacerbation). Exhaled breath was collected during tidal breathing using a standardized buffered end-tidal interface, with identical instrument settings across all measurements. Where clinically feasible, inhaled medications were withheld for 24 h prior to sampling. Detailed patient characteristics, inclusion/exclusion criteria, clinical assessments are provided in the respective publications [15,16,17,18].

This study used anthropometric data of the subjects (gender, age, BMI), smoking status, mMRC scores, results of respiratory function according to the American Thoracic Society (ATS) and European Respiratory Society (ERS) criteria (spirometry data of FEV1, FVC, FEV1/FVC and FEF75 as % of predicted values and z-score).

The protocol was reviewed and approved by the Ethics Committee of I.M. Sechenov First Moscow State Medical University (Protocol 02-23; 26 January 2023). The study adhered to the Declaration of Helsinki, was registered on ClinicalTrials.gov (https://clinicaltrials.gov/study/NCT05727852?term=NCT05727852&rank=1) (accessed on 8 April 2026) (NCT05727852), and all participants provided written informed consent.

### 4.2. PTR-TOF-MS Breath Sampling and Instrumental Setup

In this study, exhaled air samples were analyzed from all patient groups with controls during normal tidal breathing. All breath samples included in the integrated analysis were acquired using the PTR-TOF-MS platform and standardized bedside workflow described in our previous disease-specific studies [15,16,17,18]. Briefly, exhaled air was measured on an Ultra-Fast PTR-TOF 1000 instrument (Ionicon, Innsbruck, Austria) equipped with a Buffered End-Tidal Breath Sampling inlet. Participants were examined in the morning under fasting conditions after tooth brushing to minimize dietary and circadian effects. For tidal breathing, subjects breathed continuously through a disposable mouthpiece for 1 min without breath-holding, yielding approximately 12–16 respiratory cycles depending on the respiratory rate. Mass spectra were collected in full-scan mode over m/z 10-685 at 1 s time resolution using H3O+ as the reagent ion; the drift tube and inlet were maintained at 80 °C. Where applicable (BA/COPD cohort), maintenance inhaled medications were withheld for 24 h prior to sampling when clinically feasible.

### 4.3. Putative VOC Annotation

Identification of volatile organic compounds (VOCs) was performed based on library searching of full-scan PTR-TOF-MS data. Identification relied on peer-reviewed volatilomics literature, the Human Metabolome Database (HMDB), and proprietary Ionicon libraries. The search targeted ions corresponding to protonated molecules ([M + H]+) with a mass tolerance of ± 200 ppm. To increase confidence in the assignments, identification criteria included accurate mass matching, isotopic pattern evaluation, and proton affinity assessment. Given that the described approach, based on full-scan PTR-TOF-MS without tandem mass spectrometry (MS/MS) confirmation, has limited specificity for resolving isobaric and isomeric compounds, all VOC annotations should be considered tentative (putative).

### 4.4. Data Splitting, Preprocessing, and Normalization

The integrated dataset comprised PTR-TOF-MS ion intensity measurements of exhaled VOCs across five diagnostic classes: BA, COPD, CF, LAM, and healthy controls (Control). Each participant contributed a single breath sample to the analysis; accordingly, the terms “breath measurements” and “samples” are used interchangeably to denote unique participants in this study. No repeated measurements were obtained from any participant. To ensure robust model validation and to eliminate the risk of data leakage, the dataset was partitioned into a training set (70%) and an independent test set (30%) using stratified group-wise random sampling based on participant identifiers, thereby preserving the original class distribution across both subsets. Each participant was assigned exclusively to either the training or the test set. Consequently, as each participant contributed only one sample, the number of samples in each subset is equal to the number of participants.

All preprocessing steps were fitted exclusively on the training data. A custom normalizer, ControlNormalizer, was implemented to perform feature scaling based solely on the distribution of the control class. For each feature, a RobustScaler (median and interquartile range) was fitted on the control samples within the training set. This scaler was then applied to all samples (including patients) in both training and test sets. This approach mitigates the influence of disease-related variability on the normalization parameters and facilitates the interpretation of patient-group deviations relative to a healthy reference.

### 4.5. Statistical Data Analysis

#### 4.5.1. Ensemble Feature Selection

To identify stable and diagnostically informative VOC signals while mitigating overfitting and class imbalance, an ensemble feature selection strategy was implemented. From the normalized training data, 500 stratified subsamples (without replacement) were drawn, each comprising 50% of the original training observations. In each iteration, the Synthetic Minority Oversampling Technique (SMOTE) was applied to the resampled subset to balance the class distribution, addressing the underrepresentation of groups such as LAM and CF.

An XGBoost (version 3.2.0) classifier was then trained on each balanced subset. Feature importance was quantified using the built-in gain metric, which measures the average improvement in accuracy brought by a feature across all trees where it is used. The importance scores were aggregated across all 500 iterations by computing the mean importance for each feature, yielding a robust, noise-resistant ranking. The threshold for forming the final panel of features is set at the 75th percentile of the probability distribution among features with non-zero stability.

#### 4.5.2. Identifying VOC Predictors and Their Relationship to Endpoints

To determine predictive markers, a cross-validation approach was applied, during which data transformation and classifier training were conducted to evaluate predictor significance within a single model. The analysis involved repeated cross-validation, data normalization, and training gradient boosting classifiers (XGBoost, version 3.2.0) for each dataset split for binary endpoints or regressors in the case of quantitative endpoints. The effectiveness of the model and the relative importance of features were evaluated using the area under the receiver operating characteristic (ROC) curve (AUC) for classifiers, and the coefficient of determination, maximal error, explained variance, and root mean squared error for regressors were assessed.

Only VOCs were considered as potential predictors, with calibration molecules and substances with a mass-to-charge ratio (m/z) below 42 being excluded from the analysis.

#### 4.5.3. Multiclass Model Development and Validation

Multiclass classification was performed using XGBoost classifiers trained separately on each feature panel. For both panels, the number of boosting rounds was fixed at 800, and hyperparameters were optimized via grid search over the following ranges: maximum tree depth (7, 12), L1 regularization term reg_alpha (0.1, 1, 10), and L2 regularization term reg_lambda (0.1, 1, 10). Model selection was guided by repeated stratified k-fold cross-validation (10 folds, 3 repeats) on the training set, using macro-averaged ROC AUC as the optimization criterion.

The final models were evaluated on the held-out test set. Performance metrics included: macro-averaged ROC AUC in both one-vs-one (OvO) and one-vs-rest (OvR) schemes; pairwise OvO ROC AUC for all class combinations; class-wise diagnostic metrics: sensitivity, specificity, positive predictive value (PPV), and negative predictive value (NPV).

To quantify uncertainty, 95% confidence intervals (CIs) for all metrics were estimated using non-parametric bootstrap resampling (1000 iterations) on the test set predictions. Specifically, for the macro-averaged AUCs, the bootstrap procedure involved resampling test instances with replacement, recomputing the metric, and extracting the 2.5th and 97.5th percentiles of the bootstrap distribution.

#### 4.5.4. Between-Group Comparisons of VOC Abundance

Univariate comparisons of VOC abundances across the five diagnostic groups were conducted using all VOC signals of the final feature panel at the 75th percentile. For each VOC, the Kruskal–Wallis H-test was first applied to assess overall differences among groups. Upon rejection of the global null hypothesis (*p* < 0.05), all pairwise group comparisons were performed using the Mann–Whitney U test. To control the family-wise error rate arising from multiple post hoc comparisons, the Holm–Bonferroni sequential correction procedure was applied. All statistical tests were performed on the normalized data, consistent with the preprocessing pipeline described in Section 4.4.

#### 4.5.5. Network Analysis of VOC Interactions

To investigate the structure of associations between volatile organic compounds (VOCs) and their disease-specific alterations, weighted graphs were constructed separately for each diagnostic group (BA, COPD, CF, LAM, and Control) based on the selected set of 29 VOC signals.

Pairwise relationships between VOCs were quantified using a signed distance correlation approach [31,32]. Distance correlation was used to capture the strength of association, as it detects both linear and nonlinear dependencies, while the direction of monotonic association was determined using Kendall’s τ rank correlation coefficient. The signed distance correlation was defined as the product of the distance correlation and the sign of Kendall’s τ, enabling simultaneous representation of both magnitude and direction of relationships

To improve robustness and reduce sensitivity to sampling variability, a bootstrap aggregation procedure was applied within each diagnostic group. Specifically, 500 subsamples were generated without replacement, each comprising one-third of the group size (with a minimum of two observations). For each subsample, pairwise signed distance correlations were computed for all VOC pairs. The resulting correlation matrices were then aggregated across all bootstrap iterations using the median, yielding a stable estimate of the signed association structure for each group.

Graph construction was performed using the aggregated signed correlation matrices. Nodes corresponded to individual VOCs, and edges were included when the absolute value of the signed correlation exceeded 10^−6^. Edge weights were defined as the absolute value of the signed correlation, while the sign was retained for interpretation.

To characterize the structural role of each node within the network, several weighted centrality metrics were computed. Weighted degree was defined as the sum of absolute edge weights incident to a node and reflects its overall connectivity. Weighted betweenness centrality was calculated as the proportion of shortest paths (weighted by absolute edge weights) that pass through a given node, indicating its role as a bridge between network regions. Katz centrality was used as a generalized measure of influence that accounts for both direct and indirect connections; the attenuation parameter was selected adaptively as 0.9 divided by the largest eigenvalue of the weighted adjacency matrix.

To quantitatively assess the significance of disease-associated changes in network topology relative to the control group, adaptive thresholds for centrality deviations were defined. For each centrality metric, the distribution of absolute deviations was calculated as |Δ| = |metric_disease_norm − metric_control_norm| across all nodes and all disease groups (excluding the control group). Threshold values were determined as the 5th and 95th percentiles of this distribution (THRESHOLD_PERCENTILE = 0.1), corresponding to the selection of the 10% most extreme deviations (Table 3). Nodes with deviations exceeding these bounds were classified as “strong” and were highlighted accordingly in both output files and visualizations.

All centrality measures were normalized within each diagnostic group using min–max scaling to the range [0, 1], thereby eliminating scale effects associated with group size or absolute correlation values. To quantify disease-associated network remodeling, deviations in centrality were calculated for each VOC as the difference between the normalized value in the disease group and the corresponding value in the control group. Positive deviations indicate increased importance of a node in the disease network, whereas negative deviations indicate a loss of its structural role.

To identify densely connected metabolite modules, an analysis of maximal weighted cliques was performed. Since the direction of association is not essential for clustering, clique detection was conducted on graphs constructed using absolute edge weights. The threshold for edge inclusion was defined based on a moderate effect size (Cohen’s d = 0.5), converted to an equivalent correlation coefficient. A clique was defined as a fully connected subgraph, and its weight was calculated as the sum of all edge weights within the clique. For each diagnostic group, the clique with the maximum total weight was identified, and node membership within the largest clique was recorded.

Visualization of centrality deviations was performed using scatter plots, with the *y*-axis representing VOCs ordered by m/z values and the *x*-axis representing deviations from the control group. To highlight the most pronounced changes, adaptive thresholds were defined based on the 5th and 95th percentiles of the distribution of all deviations across groups and metrics. Values exceeding these thresholds were considered strong deviations and were emphasized in the visualization.

Network visualization was performed using the NetworkX library (version 3.6.1). Node size was scaled proportionally to weighted degree, while edge color indicated the sign of correlation (positive or negative). Edge thickness and transparency were proportional to the absolute value of the correlation, facilitating visual interpretation of network structure.

## 5. Conclusions

In conclusion, PTR-TOF-MS breathomics combined with compact ML-selected VOC panels shows proof-of-concept multiclass discrimination across obstructive chronic lung diseases and suggests complementary systems-level information from VOC network remodeling. Prospective, multicenter studies with larger matched cohorts are required to confirm disease-specificity and clinical utility. Future works should include participant-level validation and multicenter external cohorts.

## Figures and Tables

**Table 1:** Participants’ characteristics.

	BA	COPD	CF	LAM	Control	*p*-Value
Number of subjects	160	128	102	51	402	
Sex, % male	56 (35.0%)	111 (86.7%)	54 (52.9%)	0 (0%)	154 (38%)	<0.01
Age, years	58.4 ± 17.1	66.6 ± 10.3	25.6 ± 7.8	48.7 ± 10.9	39.1 ± 14.0	<0.01
BMI, kg/m^2^	29.3 ± 8.5	25.9 ± 5.8	19.8 ± 3.6	24.7 ± 5.1	25.0 ± 5.2	0.02
Smoking status						
Never	85 (53%)	25 (19.5%)	102 (100%)	50 (98%)	281 (69.9%)	0.06
Former	54 (33.9%)	43 (33.6%)	0	0	107 (26.6%)	<0.01
Current	21 (13.1%)	60 (46.9%)	0	1 (2%)	14 (3.5%)	<0.01
mMRC, scores	2.1 ± 0.9	2.6 ± 0.9	1.1 ± 0.8	1.5 ± 1.1	0.0 ± 0.1	<0.01
FVC % pred	78.1 ± 18.7	72.6 ± 22.3	76.0 ± 21.2	89.0 ± 18.7	98.8 ± 11.3	<0.01
FEV_1_% pred	61.9 ± 17.1	49.7 ± 21.6	57.6 ± 24.6	72.9 ± 28.6	99.2 ± 11.0	<0.01
FEV_1_/FVC, %	62.4 ± 10.0	51.1 ± 11.7	62.4 ± 13.1	63.8 ± 17.1	82.7 ± 6.2	<0.01
FEF_75_%pred	61.8 ± 27.0	52.5 ± 27.0	29.6 ± 27.8	66.3 ± 44.2	123.0 ± 52.1	<0.01

Data are presented as mean ± SD or number (%). BA: bronchial asthma; BMI: body mass index; CF: cystic fibrosis; COPD: chronic obstructive pulmonary disease; FEV1: forced expiratory volume in 1 s, FVC: forced vital capacity, FEF75: the forced expiratory flow when 75% of FVC has been exhaled; LAM: lymphangioleiomyomatosis; mMRC: Modified Medical Research Council; NA: not available; SD: standard deviation.

**Table 2:** Direct comparison differences in the presence of putative VOCs in chronic respiratory diseases vs. controls *.

m/z	VOCs Name **	BA	COPD	CF	LAM	Control
71.055	2-Pentanone, Fragments of C5-compounds	0.211 ± 0.503	0.360 ± 0.617	0.125 ± 0.299	0.058 ± 0.049	0.077 ± 0.092
73.065	Butanal or 2-butanone fragment	0.069 ± 0.070	0.166 ± 0.549	0.043 ± 0.051	0.049 ± 0.019	0.045 ± 0.021
79.054	Protonated benzene	0.022 ± 0.023	0.038 ± 0.031	0.013 ± 0.008	0.019 ± 0.028	0.014 ± 0.011
83.086	Cyclohexene	0.046 ± 0.113	0.049 ± 0.044	0.033 ± 0.214	0.034 ± 0.089	0.043 ± 0.199
85.070	Cyclopentenone	0.056 ± 0.051	0.054 ± 0.037	0.016 ± 0.005	0.023 ± 0.016	0.028 ± 0.028
95.054	Phenol	0.187 ± 0.144	0.141 ± 0.137	0.050 ± 0.086	0.284 ± 0.175	0.213 ± 0.130
97.092	Cycloheptene	0.025± 0.022	0.037 ± 0.034	0.013 ± 0.019	0.020 ± 0.028	0.017 ± 0.024
109.071	Methionol (3-methylthiopropanol)	0.032 ± 0.022	0.034 ± 0.023	0.014 ± 0.005	0.031 ± 0.028	0.029 ± 0.052
118.071	Indole	0.037 ± 0.044	0.023 ± 0.036	0.005 ± 0.009	0.060 ± 0.051	0.041 ± 0.040
149.104	Diethanolamine	0.017 ± 0.067	0.011 ± 0.010	0.014 ± 0.008	0.018 ± 0.018	0.025 ± 0.113
159.071	Thiazole/thiazolium derivatives	0.032 ± 0.173	0.060 ± 0.284	0.005 ± 0.002	0.112 ± 0.389	0.066 ± 0.340
181.014	Chlorinated amino acid derivative	0.008 ± 0.017	0.006 ± 0.006	0.009 ± 0.007	0.006 ± 0.008	0.020 ± 0.082
235.211	Terpene derivatives	0.006 ± 0.005	0.006 ± 0.005	0.012 ± 0.027	0.011 ± 0.008	0.015 ± 0.056

* The Kruskal–Wallis test was used; data are presented as area comparisons (peak area for the target ion expressed as count per second), *p* < 0.001 for all comparison differences (full pairwise *p*-values are presented in Table S5; full annotation of the most significant VOCs is presented in Table S6.); ** the putative chemical was identified using Ionicon libraries, the Human Metabolome Database and literature data; BA: bronchial asthma; COPD: chronic obstructive pulmonary disease; CF: cystic fibrosis; LAM: lymphangioleiomyomatosis; m/z: mass to charge; NA: not available.

**Table 3:** Adaptive threshold values for centrality deviations based on percentile distribution.

Centrality Metric	Lower Threshold (5th Percentile)	Upper Threshold (95th Percentile)
Weighted Degree	−0.295	+0.448
Betweenness	−0.426	+0.271
Eigenvector	−0.306	+0.449
Katz	−0.303	+0.446

## Data Availability

All data supporting the findings of this study are provided within the article and/or the Supplementary Materials. Requests for additional information may be addressed to the corresponding author.

## Supplementary Materials

The following supporting information can be downloaded at: https://www.mdpi.com/article/10.3390/ijms27083483/s1.

## Abbreviations

The following abbreviations are used in this manuscript:

PTR-TOF-MS	proton-transfer reaction time-of-flight mass spectrometry
eVOCs	exhaled volatile organic compounds
BA	bronchial asthma
COPD	chronic obstructive pulmonary disease
CF	cystic fibrosis
LAM	lymphangioleiomyomatosis
ATS	the American Thoracic Society
ERS	the European Respiratory Society
AUC	area under the curve
BMI	body mass index
FEV_1_	forced expiratory volume in 1 s
FVC	forced vital capacity
FEF_75_	forced expiratory flow when 75% of FVC has been exhaled
mMRC	Modified Medical Research Council
OvO	one-vs-one
OvR	one-vs-rest
