# Supplementary material for: Cross-Disease Breathomics by PTR-TOF-MS: Multiclass Machine Learning and Network Remodeling Across Asthma, COPD, Cystic Fibrosis, and Lymphangioleiomyomatosis
Source: Int J Mol Sci. 2026 Apr 13;27(8):3483. doi: 10.3390/ijms27083483 (PMC13116732; doi:10.3390/ijms27083483)
Supplement: Supplementary file 1 [file ijms-27-03483-s001.zip › Supplementary materials_red.pdf]

Cross-Disease Breathomics by PTR-TOF-MS: Multiclass Machine Learning and Network Remodeling across Asthma, COPD, Cystic Fibrosis, and Lymphangioleiomyomatosis

Malika Mustafina [1,2,3,*], Artemiy Silantyev [4], Aleksandr Suvorov [4], Stanislav Krasovskiy [2], Marina Makarova [2,5], Alexander Chernyak [2], Olga Suvorova [6], Anna Shmidt [6], Daria Gognieva [1,3,4], Aleksandra Bykova [1,3], Nana Gogiberidze [1], Andrei Akselrod [1], Andrey Belevskiy [5], Sergey Avdeev [2,6], Vladimir Betelin [3], Abram Syrkin [1] and Philipp Kopylov [1,3,4]

1 Department of Cardiology, Functional and Ultrasound Diagnostics, I.M. Sechenov First Moscow State Medical University (Sechenov University), 119048 Moscow, Russia; gognieva_d_g@staff.sechenov.ru (D.G.); aabykowa@yandex.ru (A.B.); nana10.11@mail.ru (N.G.); akselrod_a_b@student.sechenov.ru (A.A.); syrkin_a_l@staff.sechenov.ru (A.S.);

2 Pulmonology Research Institute under the Federal Medical and Biological Agency of Russia, 115682 Moscow, Russia; sa_krasovsky@mail.ru (S.K.); achi2000@mail.ru (A.C.)

3 Research Institute for Systemic Analysis of the Russian Academy of Sciences, 117218 Moscow, Russia; betelin@niisi.msk.ru

4 World-Class Research Center "Digital Biodesign and Personalized Healthcare", I.M. Sechenov First Moscow State Medical University (Sechenov University), 119048 Moscow, Russia; artsilan@gmail.com (A.S.); suvorov_a_yu_1@staff.sechenov.ru (A.S.); kopylov_f_yu@staff.sechenov.ru (P.K.)

5 N.I. Pirogov Russian National Research Medical University, 1 Ostrovityanova str., Bldg. 6, 117513 Moscow, Russia; pulmobas@yandex.ru (A.B.); mma123@list.ru (M.M.)

6 Pulmonology Department, I.M. Sechenov First Moscow State Medical University (Sechenov University), 119048 Moscow, Russia; olga.a.suvorova@mail.ru (O.S.); a_e_schmidt@mail.ru (A.S.); avdeev_s_n@staff.sechenov.ru (S.A.)

* Correspondence: mustafina_m_kh@staff.sechenov.ru; Tel.: +7-9164785942

Table S1. Composition of the integrated dataset and stratified train/test split (breath measurements).

Group	Total participants (n=843)	Train (n=590)	Test (n=253)
Control	402 (47.6%)	281 (47.6%)	121 (47.7%)
BA	160 (19.0%)	112 (19.0%)	48 (19.0%)
COPD	128 (15.3%)	90 (15.3%)	38 (15.0%)
CF	102 (12.1%)	71 (12.0%)	31 (12.3%)
LAM	51 (6.0%)	36 (6.1%)	15 (6.0%)

BA: bronchial asthma; COPD: chronic obstructive pulmonary disease; CF: cystic fibrosis; LAM: lymphangioleiomyomatosis

Table S2. Feature selection stability probabilities (stability selection, n = 1000 iterations).

Feature (m/z or covariate)	Probability of selection
83.08564	1
95.082	1

95.05365	1
97.09225	1
96.05719	1
159.0712	1
97.10352	1
Age	0.999
235.21074	0.999
71.05515	0.998
71.08162	0.998
181.01353	0.996
149.10438	0.989
85.09638	0.986
85.0697	0.985
105.93981	0.985
BMI	0.958
73.06527	0.954
107.9565	0.939
79.05395	0.938
109.07056	0.934
55.03962	0.915
109.09555	0.914
119.95525	0.893
103.07641	0.893
118.0709	0.891
58.94678	0.882
45.99156	0.879
329.83984	0.87
77.05868	0.867
47.04044	0.827
119.07616	0.825
113.13138	0.814
93.06971	0.811
355.07362	0.803
108.96676	0.796
144.91809	0.789
137.13656	0.769
371.09815	0.748
135.11621	0.742
356.07436	0.736
49.00474	0.694
72.05698	0.692
63.02014	0.682
75.04626	0.669
123.05295	0.665
153.13312	0.665
138.13952	0.655
48.04484	0.637

357.07278	0.635
373.0848	0.629
171.17475	0.622
374.08458	0.609
123.95023	0.604
123.11694	0.603
53.03764	0.6
58.95657	0.586
64.02823	0.579
115.10493	0.54
115.07041	0.528
44.9912	0.525
372.10003	0.521
48.00325	0.509
86.10319	0.459
99.11442	0.444
101.05758	0.422
99.04708	0.421
99.06579	0.4
99.08365	0.387
87.07784	0.378
110.97561	0.377
101.0412	0.376
87.05691	0.372
70.07543	0.37
69.07288	0.359
58.07289	0.357
74.04733	0.35
330.84532	0.332
111.11377	0.33
49.99381	0.311
331.8501	0.306
58.04341	0.286
94.05909	0.286
56.05216	0.266
76.04996	0.248
103.95537	0.248
91.05658	0.203
117.08769	0.189
91.04632	0.164
61.03327	0.157
60.05513	0.153
107.08925	0.146
55.93497	0.13
133.10377	0.126
51.03858	0.124
93.96037	0.121

82.07531	0.117
121.09658	0.116
44.01798	0.109
126.91063	0.103
81.07274	0.098
105.06843	0.086
59.05849	0.084
Gender	0.076
203.94814	0.072
44.04878	0.066
204.9509	0.062
57.06893	0.059
57.0367	0.052
62.02928	0.052
65.05831	0.049
90.95121	0.041
65.02656	0.041
127.14426	0.037
89.05796	0.037
90.05956	0.034
67.05657	0.022
143.13048	0.019
129.08657	0.008
68.06282	0.005
43.01272	0.002
43.04723	0
Smoking status	0

Confusion matrices for the training and test datasets are presented in Table 5. On the training set, both models demonstrate near-perfect classification, reflecting a high capacity to fit the training data. On the independent test set, a decrease in accuracy is observed, which is expected and indicates the absence of severe overfitting.

Table S3A. Error matrix of the full model on the test sample

True	BA	CF	COPD	Control	LAM
BA	21	1	1	4	2
CF	0	21	0	0	0
COPD	14	0	19	2	0
Control	18	2	5	96	0
LAM	3	0	0	6	7

Table S3B. Error matrix of the VOC model on the test sample

True	BA	CF	COPD	Control	LAM
BA	24	0	1	4	0
CF	0	24	1	4	0

COPD	17	1	13	3	1
Control	16	1	8	93	3
LAM	3	0	1	6	6

Rows are true classes, columns are predicted classes. Diagonal elements (in bold) are correct classifications.

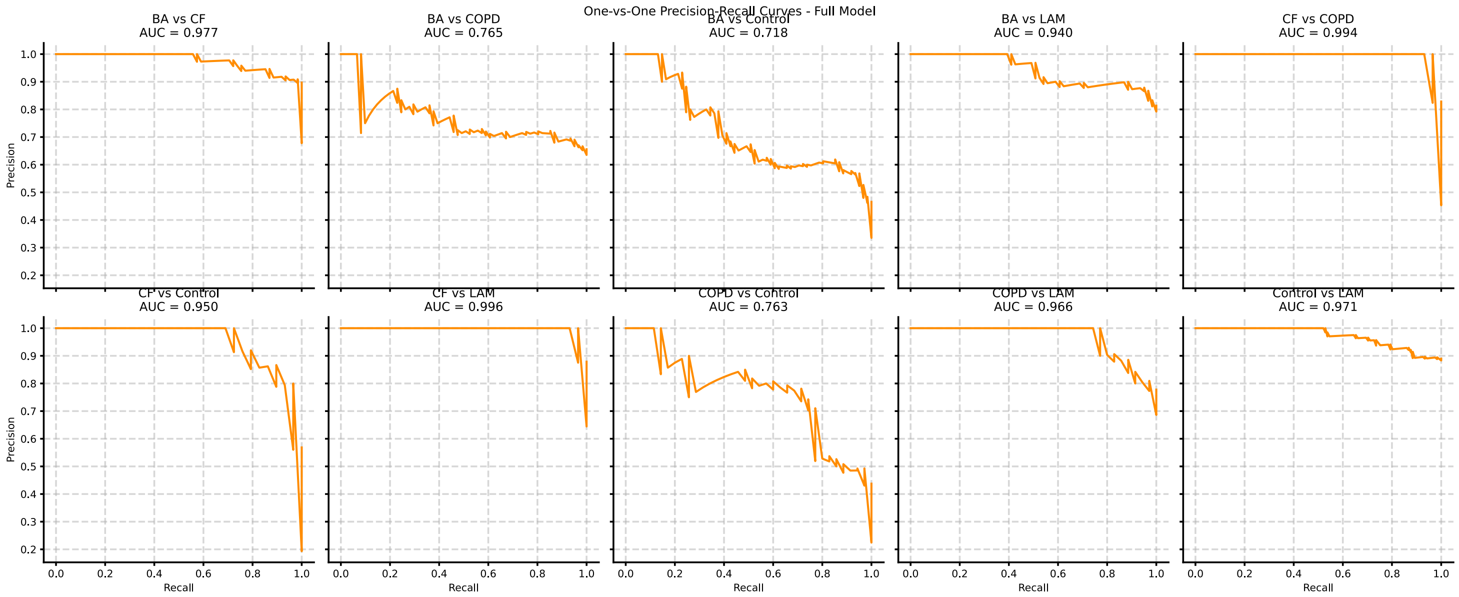

Figure S1A. The Precision-Recall curves for full models (VOCs + covariants).

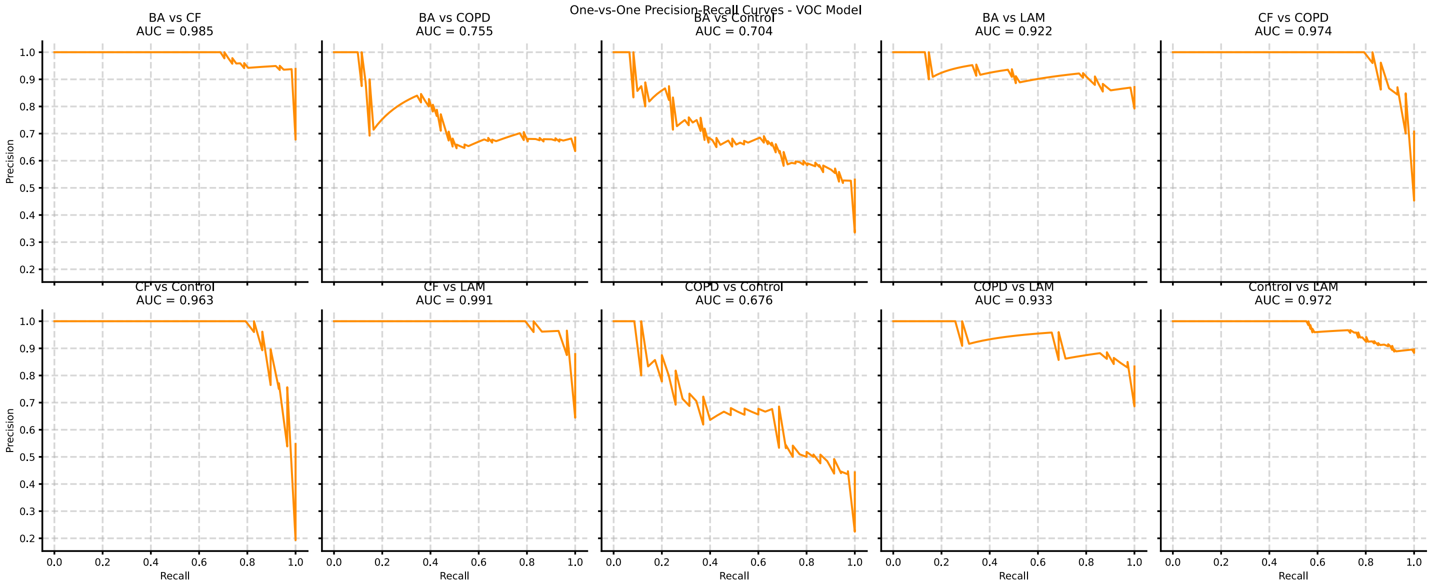

Figure S1B. The Precision-Recall curves for VOCs-only models.

Table S4. Key metrics for models quality

Class comparison	Model	AUC (CI)	Se (CI)	Sp (CI)	PPV (CI)	NPV (CI)

**BA vs CF**	Full	**0.956** (0.907-0.991)	0.528 (0.407-0.651)	1.000 (1.000-1.000)	1.000 (1.000-1.000)	0.500 (0.381-0.624)
	VOC	**0.973** (0.934-1.000)	0.610 (0.500-0.723)	1.000 (1.000-1.000)	1.000 (1.000-1.000)	0.547 (0.422-0.677)
**BA vs COPD**	Full	**0.660** (0.536-0.762)	0.246 (0.138-0.350)	0.913 (0.808-1.000)	0.832 (0.643-1.000)	0.409 (0.306-0.517)
	VOC	**0.629** (0.510-0.738)	0.148 (0.066-0.244)	0.971 (0.906-1.000)	0.896 (0.700-1.000)	0.394 (0.295-0.500)
**BA vs Control**	Full	**0.844** (0.788-0.899)	0.524 (0.403-0.647)	0.829 (0.752-0.901)	0.608 (0.475-0.740)	0.776 (0.707-0.845)
	VOC	**0.847** (0.792-0.900)	0.601 (0.475-0.717)	0.861 (0.797-0.922)	0.685 (0.561-0.815)	0.811 (0.748-0.874)
**BA vs LAM**	Full	**0.804** (0.681-0.911)	0.525 (0.397-0.639)	0.808 (0.586-1.000)	0.912 (0.814-1.000)	0.311 (0.175-0.439)
	VOC	**0.794** (0.645-0.919)	0.608 (0.492-0.715)	0.751 (0.533-0.938)	0.902 (0.802-0.976)	0.336 (0.186-0.487)
**CF vs COPD**	Full	**0.994** (0.980-1.000)	0.727 (0.558-0.882)	1.000 (1.000-1.000)	1.000 (1.000-1.000)	0.816 (0.699-0.923)
	VOC	**0.975** (0.937-0.997)	0.758 (0.600-0.893)	1.000 (1.000-1.000)	1.000 (1.000-1.000)	0.834 (0.721-0.931)
**CF vs Control**	Full	**0.986** (0.966-0.997)	0.722 (0.556-0.860)	0.991 (0.971-1.000)	0.953 (0.842-1.000)	0.937 (0.891-0.973)
	VOC	**0.988** (0.969-0.999)	0.759 (0.607-0.898)	1.000 (1.000-1.000)	1.000 (1.000-1.000)	0.946 (0.907-0.977)
**CF vs LAM**	Full	**0.990** (0.964-1.000)	0.726 (0.554-0.873)	1.000 (1.000-1.000)	1.000 (1.000-1.000)	0.669 (0.490-0.833)
	VOC	**0.983** (0.950-1.000)	0.761 (0.603-0.902)	1.000 (1.000-1.000)	1.000 (1.000-1.000)	0.698 (0.522-0.870)
**COPD vs Control**	Full	**0.918** (0.867-0.957)	0.572 (0.388-0.743)	0.958 (0.917-0.992)	0.799 (0.622-0.954)	0.885 (0.828-0.938)

	VOC	**0.888** (0.830-0.934)	0.428 (0.257-0.600)	0.934 (0.886-0.974)	0.651 (0.462-0.830)	0.849 (0.787-0.906)
**COPD vs LAM**	Full	**0.921** (0.841-0.984)	0.574 (0.414-0.739)	1.000 (1.000-1.000)	1.000 (1.000-1.000)	0.517 (0.339-0.681)
	VOC	**0.889** (0.775-0.976)	0.435 (0.284-0.603)	0.942 (0.806-1.000)	0.942 (0.818-1.000)	0.432 (0.273-0.597)
**Control vs LAM**	Full	**0.800** (0.714-0.880)	0.571 (0.476-0.660)	0.873 (0.688-1.000)	0.971 (0.927-1.000)	0.213 (0.119-0.317)
	VOC	**0.808** (0.709-0.884)	0.588 (0.500-0.676)	0.812 (0.600-1.000)	0.960 (0.914-1.000)	0.208 (0.113-0.313)

OvO Macro AUC denotes the macro-averaged area under the ROC curve in the one-vs-one framework. Se — sensitivity; Sp — specificity; PPV — positive predictive value; NPV — negative predictive value. All binary metrics were computed in a one-vs-rest setting using fixed thresholds determined on the training set based on Youden's index. Ninety-five percent confidence intervals were estimated via nonparametric bootstrapping (500 iterations).

Table S5. Pairwise p-values for comparative analysis of VOC levels in the study groups.

VOC (m/z)	Ctr vs LAM	Ctr vs BA	Ctr vs COPD	Ctr vs CF	LAM vs BA	LAM vs COPD	LAM vs CF	BA vs COPD	BA vs CF	COPD vs CF
**97.1035**	1.000	<0.001	<0.001	<0.001	0.033	<0.001	<0.001	<0.001	<0.001	<0.001
**97.0923**	1.000	<0.001	<0.001	<0.001	0.032	<0.001	<0.001	<0.001	<0.001	<0.001
**96.0572**	0.005	0.06	<0.001	<0.001	0.001	<0.001	<0.001	0.024	<0.001	<0.001
**95.082**	0.006	0.002	<0.001	<0.001	1.000	<0.001	<0.001	0.001	<0.001	<0.001
**95.0537**	0.006	0.002	<0.001	<0.001	1.000	<0.001	<0.001	0.001	<0.001	<0.001
**85.0964**	0.273	<0.001	<0.001	<0.001	1.000	<0.001	<0.001	0.321	<0.001	<0.001
**85.0697**	0.281	<0.001	<0.001	<0.001	1.000	<0.001	<0.001	0.326	<0.001	<0.001
**83.0856**	0.095	<0.001	<0.001	0.001	1.000	<0.001	<0.001	0.001	<0.001	<0.001
**79.054**	0.059	<0.001	<0.001	0.003	0.067	<0.001	<0.001	1.000	<0.001	<0.001
**77.0587**	0.597	0.014	<0.001	<0.001	0.06	0.002	<0.001	0.033	<0.001	<0.001
**73.0653**	0.057	<0.001	<0.001	<0.001	0.003	<0.001	<0.001	0.001	<0.001	<0.001
**71.0816**	0.966	<0.001	<0.001	0.343	1.000	<0.001	0.589	0.001	<0.001	<0.001

VOC (m/z)	Ctr vs LAM	Ctr vs BA	Ctr vs COPD	Ctr vs CF	LAM vs BA	LAM vs COPD	LAM vs CF	BA vs COPD	BA vs CF	COPD vs CF
**71.0551**	0.96	<0.001	<0.001	0.344	1.000	<0.001	0.595	0.001	<0.001	<0.001
**58.9468**	0.317	0.002	0.003	<0.001	0.019	0.012	0.044	0.63	<0.001	<0.001
**55.0396**	0.003	0.001	0.054	0.683	0.981	0.754	0.007	0.958	0.007	0.133
**47.0404**	0.012	0.098	0.261	0.001	0.001	0.098	0.872	0.023	<0.001	0.069
**45.9916**	0.026	<0.001	<0.001	0.01	1.000	<0.001	0.74	0.929	<0.001	<0.001
**329.84**	<0.001	<0.001	<0.001	<0.001	1.000	<0.001	0.011	0.065	<0.001	<0.001
**235.211**	0.005	<0.001	<0.001	0.574	1.000	<0.001	0.003	0.497	<0.001	<0.001
**181.014**	0.72	<0.001	0.003	0.008	0.004	0.075	0.006	0.498	<0.001	<0.001
**159.071**	<0.001	0.923	0.583	0.101	1.000	<0.001	<0.001	0.583	0.151	0.583
**149.104**	<0.001	0.036	0.323	<0.001	1.000	<0.001	0.323	0.558	<0.001	<0.001
**119.955**	0.354	<0.001	<0.001	<0.001	1.000	<0.001	0.015	0.743	<0.001	<0.001
**118.071**	0.005	0.007	<0.001	<0.001	1.000	<0.001	<0.001	1.000	<0.001	<0.001
**109.096**	0.142	0.001	<0.001	<0.001	0.702	0.111	<0.001	0.108	<0.001	<0.001
**109.071**	0.2	0.001	<0.001	<0.001	0.493	0.055	<0.001	0.109	<0.001	<0.001
**107.957**	0.924	<0.001	<0.001	<0.001	1.000	<0.001	0.001	0.123	<0.001	<0.001
**105.94**	0.003	0.002	0.009	0.649	0.003	0.003	1.000	0.893	0.055	0.079
**103.076**	0.086	0.003	0.418	<0.001	0.974	0.378	<0.001	0.18	<0.001	<0.001

Global differences between groups were assessed using the Kruskal-Wallis test (for all variables). Pairwise p-values were adjusted for multiple comparisons using the Benjamini-Hochberg test.

Table S6. Putative annotation of the most significant VOCs

Putative VOC name	Measured m/z	Theoretical mass ($[M+H]^+$)	Error (ppm)	Isotopic composition	Molecular formula
Phenol	95.05365	95.04969	41.7 ppm	M: $^{12}C_6{}^1H_7{}^{16}O^+$ (100%)<br>M+1: ~6.6% (^{13}C contribution)<br>M+2: ~0.2–0.4% (^{18}O contribution)	C_6H_6O (ion: $C_6H_7O^+$)
Cyclohexene	83.08564	83.08608	-5.3 ppm	M: $^{12}C_6{}^1H_{11}{}^+$ (100%)<br>M+1: ~6.6% (^{13}C contribution)	C_6H_{10} (ion: $C_6H_{11}{}^+$)

				M+2: <0.1%	
Cycloheptene	97.09225	97.09664	-45.2 ppm	M: $^{12}C_6{}^1H_{13}{}^{16}O^+$ (100%)<br>M+1: ~6.6% (^{13}C contribution)<br>M+2: ~0.2–0.4% (^{18}O contribution)	$C_6H_{12}O$ (ion: $C_6H_{13}O^+$)
Thiazole / thiazolium derivatives	159.0712	159.06841	-1.49	M: $^{12}C_7{}^1H_{10}{}^{14}N^{16}O^{32}S_2{}^+$ (100%)<br>M+1: ~8–9% (^{13}C, minor ^{15}N, ^{33}S)<br>M+2: ~8–10% (mainly ^{34}S contribution)	$C_7H_9NOS_2$ (ion: $C_7H_{10}NOS_2{}^+$)
Terpene	235.21074	235.20110	41.0 ppm	M: $^{12}C_{15}{}^1H_{27}{}^{16}O_2{}^+$ (100%)<br>M+1: ~16.5% (^{13}C contribution)<br>M+2: ~1–2% (^{18}O and $^{13}C_2$)	$C_{15}H_{26}O_2$ (ion: $C_{15}H_{27}O_2{}^+$)
2-Pentanone, Fragments of C5-compounds	71.05515	71.04969	76.8 ppm	M: $^{12}C_4{}^1H_7{}^{16}O^+$ (100%)<br>M+1: ~4.4% (mainly ^{13}C contribution)<br>M+2: ~0.2–0.3% (^{18}O contribution)	C_4H_6O (ion: $C_4H_7O^+$)
chlorinated amino acid derivative	181.01353	181.01419	-3.6 ppm	M: $^{12}C_5{}^1H_8{}^{35}Cl^{14}N^{16}O_4{}^+$ (100%)<br>M+1: ~5.8% (mainly ^{13}C, minor ^{15}N)<br>M+2: ~32–33% (mainly ^{37}Cl contribution)	$C_5H_7ClNO_4$ (ion: $C_5H_8ClNO_4{}^+$)
Diethanolamine	149.10438	149.10519	-5.5 ppm	M: $^{12}C_6{}^1H_{15}{}^{14}N^{16}O_3{}^+$ (100%)<br>M+1: ~7.0% (mainly ^{13}C, minor ^{15}N contribution)<br>M+2: ~0.7–0.8% (mainly ^{18}O and two ^{13}C atoms)	$C_6H_{14}NO_3$ (ion: $C_6H_{15}NO_3{}^+$)
Cyclopentenone	85.0697	85.06534	51.3 ppm	M: $^{12}C_5{}^1H_9{}^{16}O^+$ (100%)<br>M+1: ~5.5% (mainly ^{13}C contribution)<br>M+2: ~0.2–0.3% (mainly ^{18}O contribution)	C_5H_8O (ion: $C_5H_9O^+$)
Butanone (methyl ethyl ketone, MEK)	73.06527	73.06479	6.6 ppm	M: $^{12}C_4{}^1H_9{}^{16}O^+$ (100%)<br>M+1: ~4.4% (mainly ^{13}C contribution)<br>M+2: ~0.2–0.3% (mainly ^{18}O contribution)	C_4H_8O (ion: $C_4H_9O^+$)
Benzene	79.05395	79.05478	-10.4 ppm	M: $^{12}C_6{}^1H_7{}^+$ (100%)<br>M+1: ~6.6% (^{13}C contribution)<br>M+2: <0.1%	C_6H_6 (ion: $C_6H_7{}^+$)

Methionol (3-methylthiopropanol)	109.07056	109.06871	17.0 ppm	M: $^{12}C_4{}^1H_{13}{}^{16}O^{32}S^+$ (100%)<br>M+1: ~4.5% (mainly ^{13}C contribution, minor ^{33}S)<br>M+2: ~4.4–4.8% (mainly ^{34}S contribution)	$C_4H_{12}OS$ (ion: $C_4H_{13}OS^+$)
Indole/Methyl Indole derivatives	118.0709	118.06905	15.7 ppm	M: $^{12}C_8{}^1H_8{}^{14}N^+$ (100%)<br>M+1: ~9.2% (mainly ^{13}C, minor ^{15}N)<br>M+2: <0.5%	C_8H_7N (ion: $C_8H_8N^+$)

Table S8. Betweenness centrality deviations for COPD, BA, CF, and LAM

Node (m/z)	Class	Δ Btw	Direction
47.04044	COPD	+0.852*	strengthening
235.21074	CF	+0.926*	strengthening
159.0712	BA	+0.370*	strengthening
159.0712	LAM	+0.370*	strengthening
181.01353	COPD	−1.000*	weakening
181.01353	CF	−1.000*	weakening
181.01353	LAM	−0.833*	weakening
149.10438	COPD	−0.426*	weakening
149.10438	CF	−0.426*	weakening
149.10438	LAM	−0.426*	weakening
71.05515	CF	+0.292*	strengthening

Table S9. Eigenvector centrality deviations for COPD, BA, CF, and LAM

Node (m/z)	Class	Δ Eig	Direction
159.0712	CF	+0.761*	strengthening
149.10438	LAM	+0.681*	strengthening
149.10438	COPD	+0.583*	strengthening
149.10438	CF	+0.471*	strengthening
96.05719	CF	+0.451*	strengthening
45.99156	CF	+0.440*	strengthening
71.05515 / 71.08162	COPD	−0.418*	weakening
71.05515 / 71.08162	CF	−0.324*	weakening
119.95525	CF	−0.343*	weakening

Centrality values were normalized within each group (Min–Max scaling) and deviations from controls were computed. BA: bronchial asthma; COPD: chronic obstructive pulmonary disease; CF: cystic fibrosis; LAM: lymphangioleiomyomatosis; *m/z:* mass to charge.

Weighted Correlation Graph — Class: BA

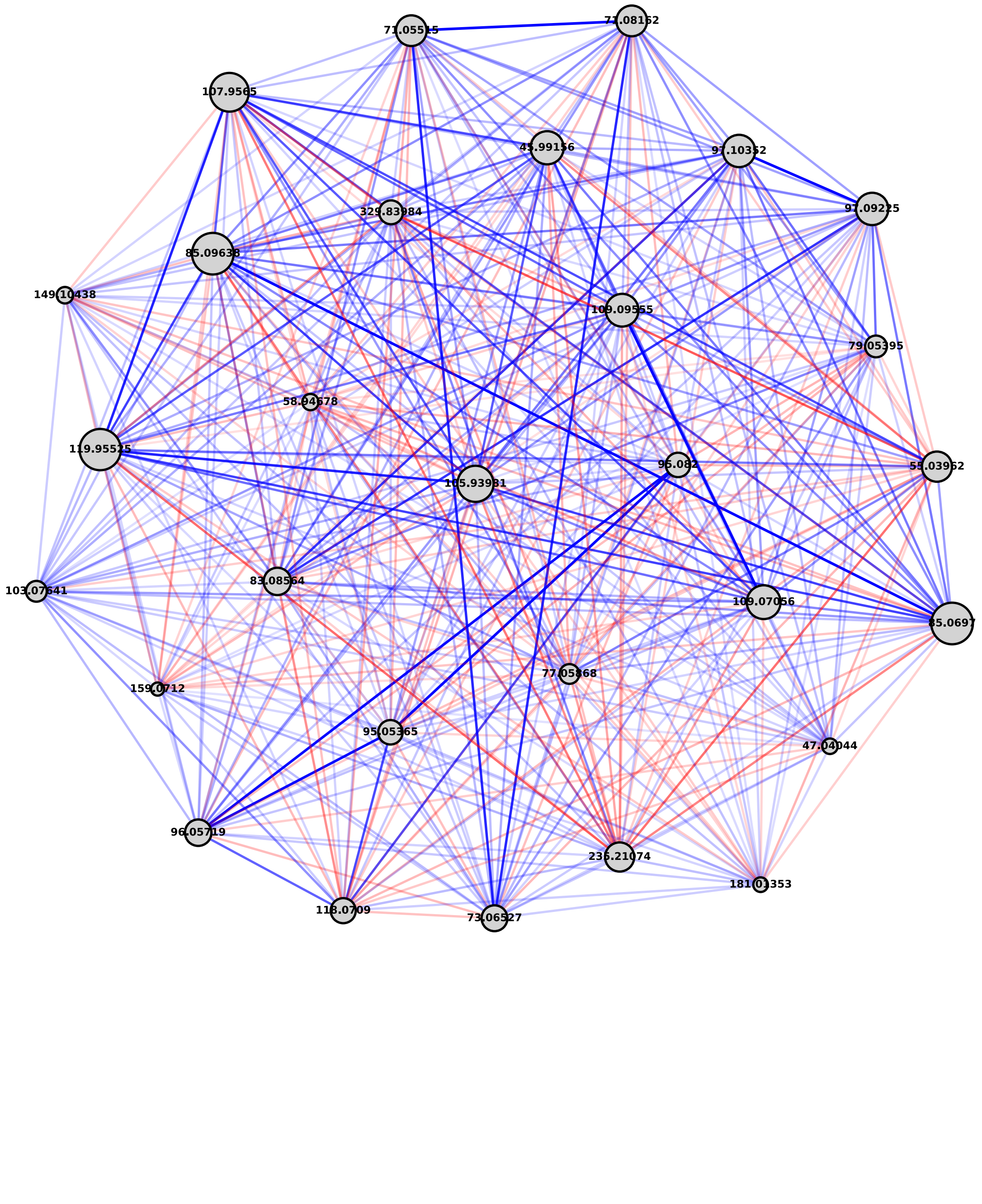

Weighted Correlation Graph — Class: CF

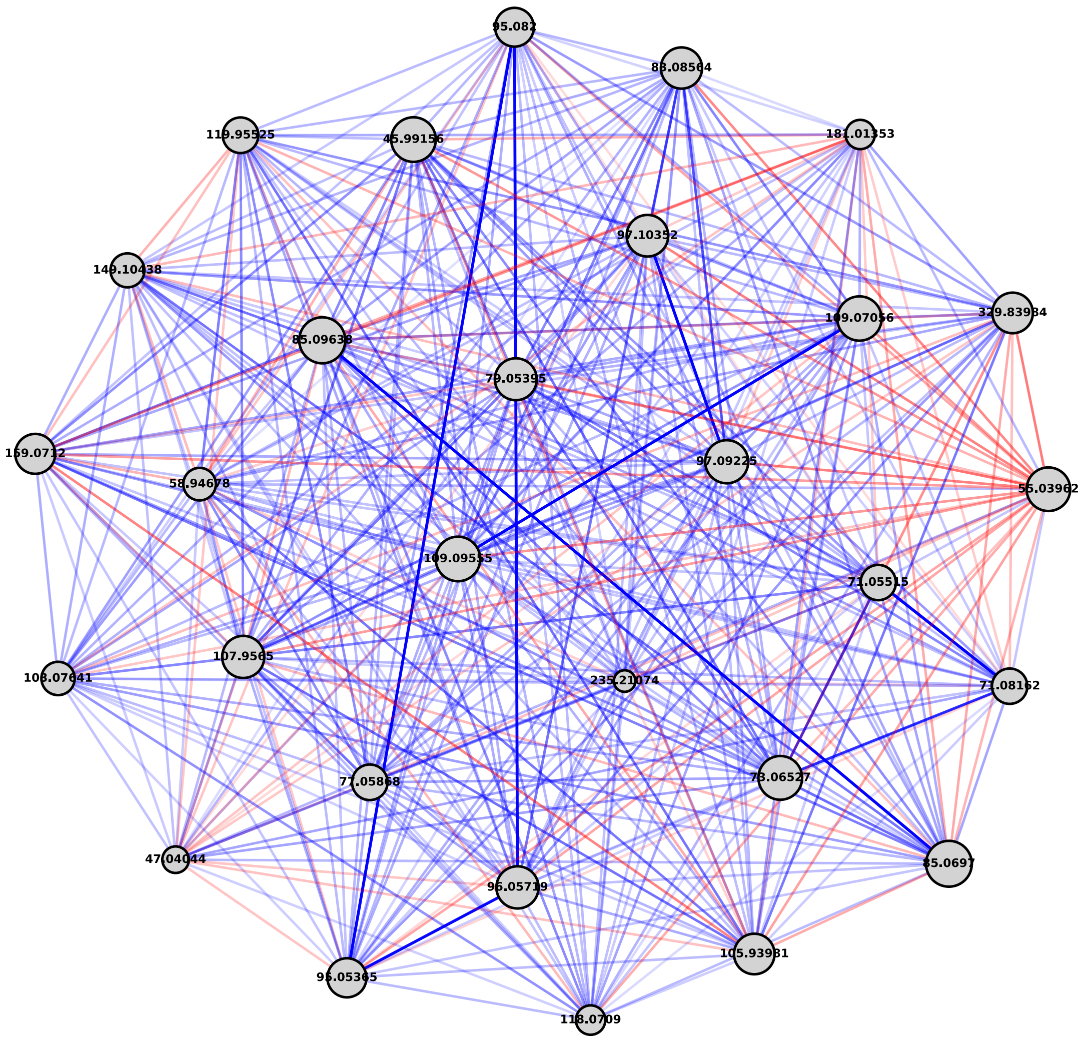

Weighted Correlation Graph — Class: Control

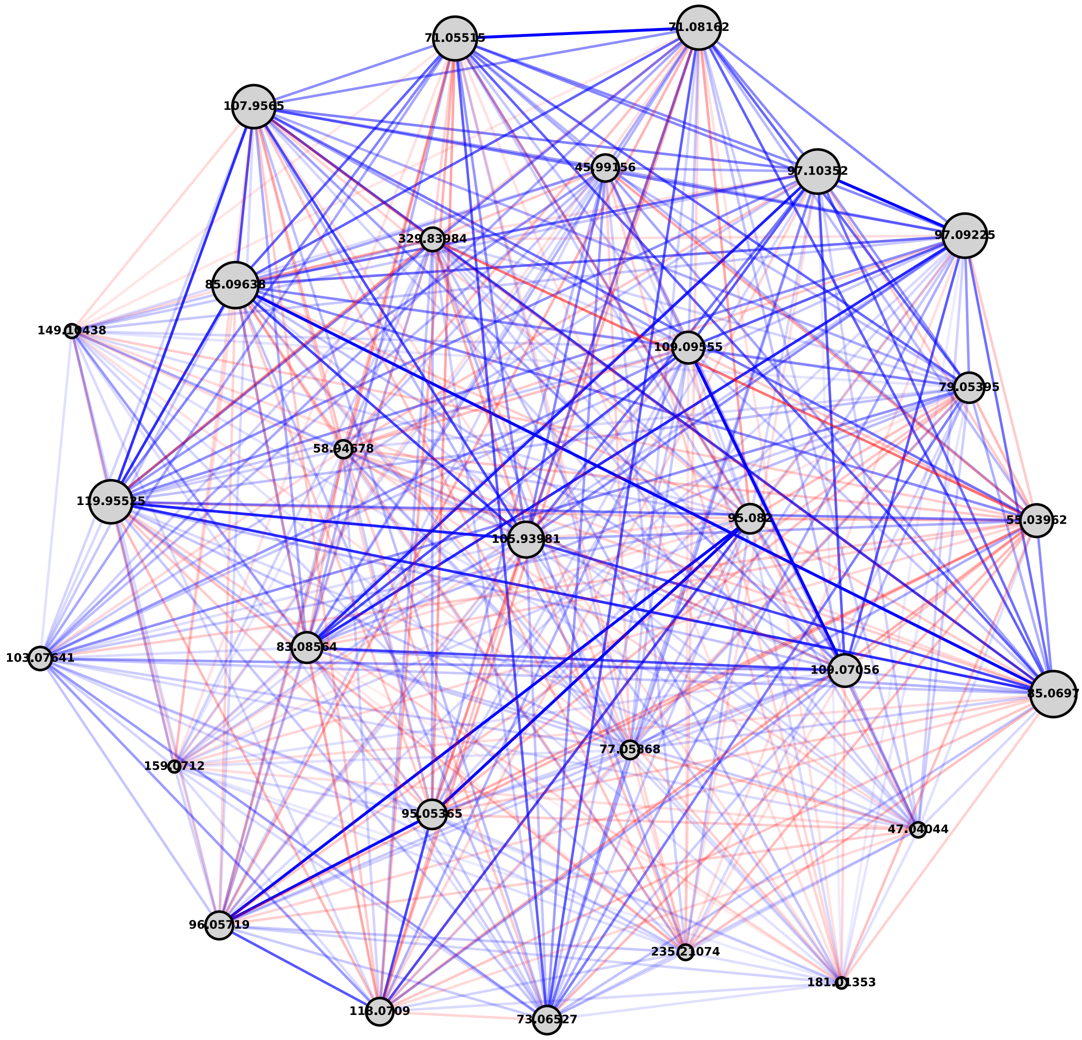

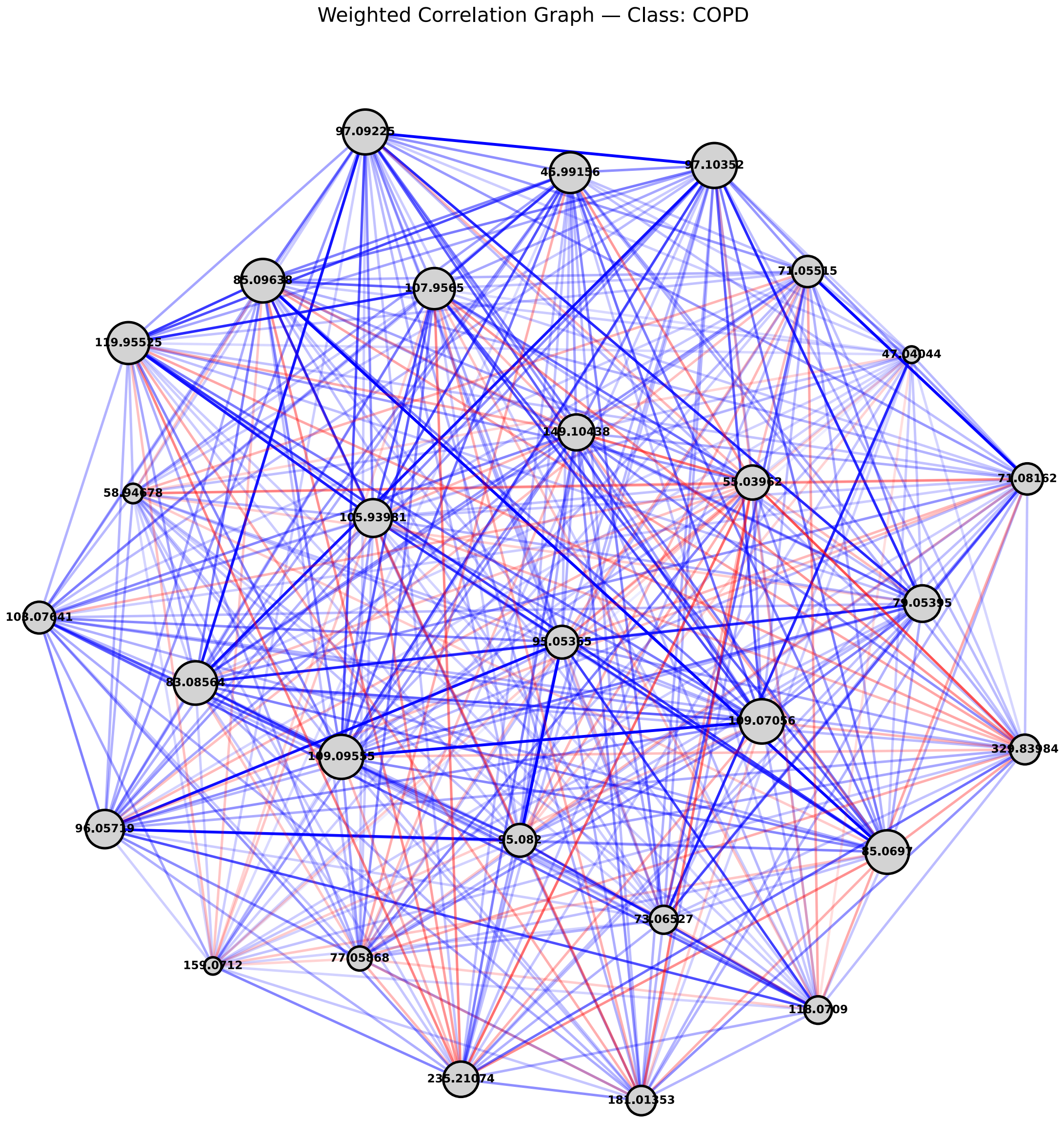
Weighted Correlation Graph — Class: COPD
97.09225
97.10352
45.99156
71.05515
47.04044
85.09639
107.9565
119.95525
149.10438
55.03962
71.08162
58.94678
105.93981
79.05395
108.07641
95.05365
83.08564
109.07056
329.83984
109.09535
96.05739
95.082
85.0697
73.06527
159.0712
77.05868
118.0709
235.21074
181.01353

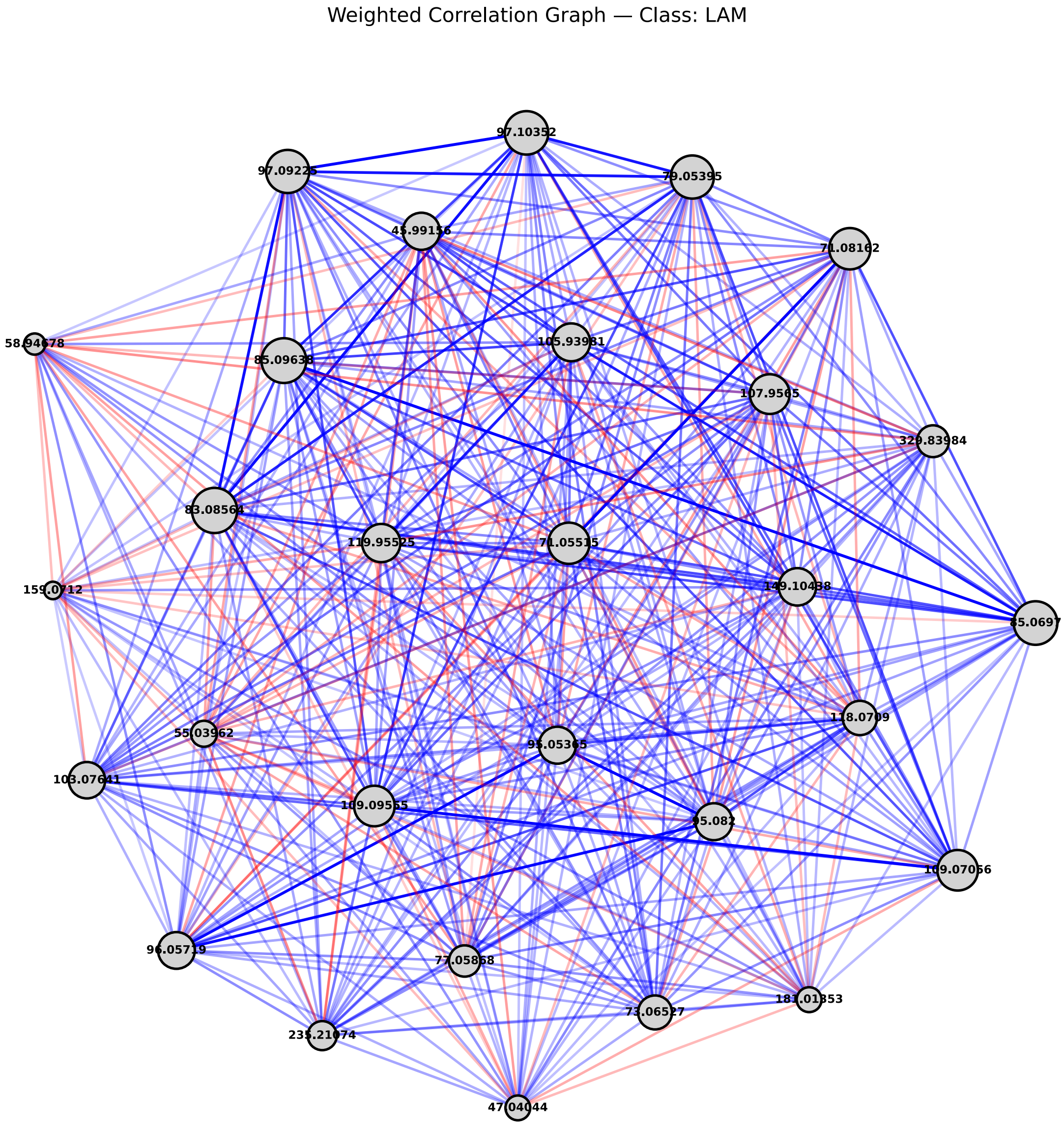

Figure S2. Weighted correlation graphs of five classes: BA, COPD, CF, LAM and Control group. Nodes represent VOC features (m/z), and edges represent pairwise associations quantified using signed distance correlation. Edge thickness corresponds to the magnitude of the correlation, and color indicates its sign (blue is positive, red is negative). Graphs are shown without thresholding to allow inspection of overall edge distributions.

Results of comparison of normalization methods

A comparative analysis of feature selection stability was conducted under three different data scaling approaches:

ControlNormalizer — normalization using RobustScaler, with parameters (median and interquartile range) estimated exclusively from the subset of healthy controls within each resampling iteration.

StandardScaler (Full Data) — standard scaling to zero mean and unit variance, with parameters estimated from the entire training dataset without isolating a control group.

RobustScaler (Full Data) — robust scaling based on the median and interquartile range, with parameters likewise estimated from the entire training dataset without isolating a control group.

For each method, a Stability Selection procedure was performed using identical settings: 250 resampling iterations, subsampling of 50% of the data, class balancing via SMOTE, and Elastic Net with internal 3-fold cross-validation to tune the regularization parameter C∈[0.01,0.1,1,10]. For each feature, the selection probability was computed as the proportion of iterations in which the model coefficient was non-zero.

The comparison included: computation of Pearson correlations between vectors of selection probabilities across pairs of methods; determination of the intersection of the top 25% feature sets (features with selection probability ≥ the 75th percentile); visualization of pairwise relationships between selection probabilities.

Table S10. Correlations of selection stability probabilities between normalization methods

**Methods**	**Pearson correlation (r)**
ControlNormalizer – RobustScaler (Full Data)	**0.869**
StandardScaler – RobustScaler (Full Data)	0.290
ControlNormalizer – StandardScaler (Full Data)	0.202

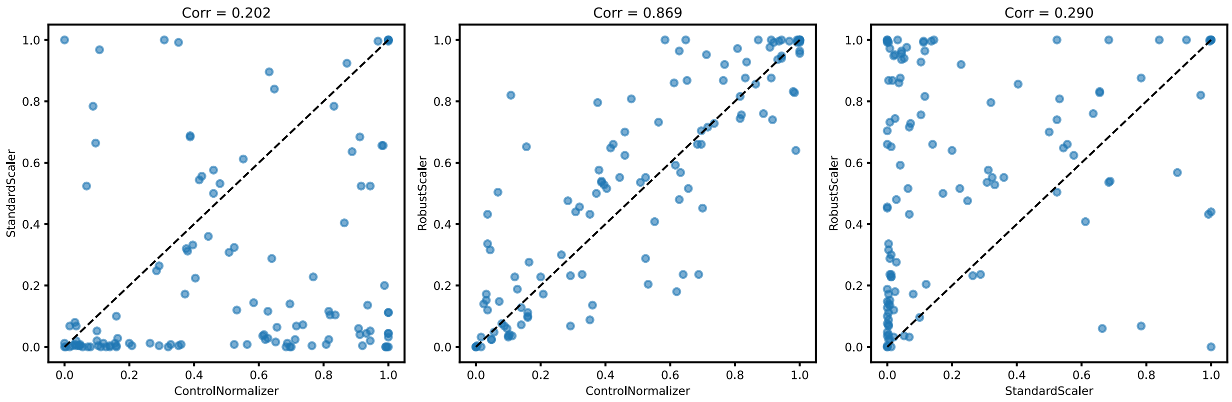

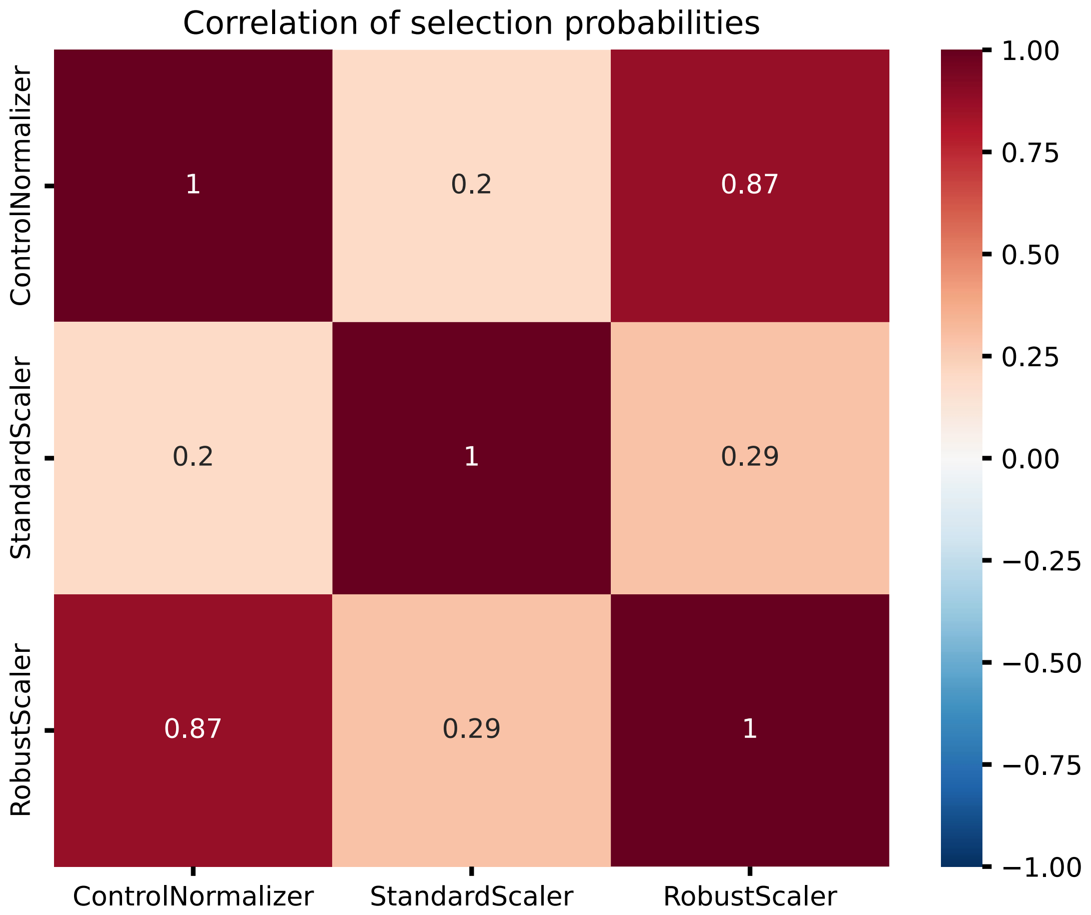

Table S11. Top 25% feature set intersection between normalization methods

**Methods**	**Number of features in the top 25%**
ControlNormalizer	30
StandardScaler (Full Data)	29
RobustScaler (Full Data)	30

**Intersection**	**Number of common features**	**Share of ControlNormalizer (%)**
ControlNormalizer ∩ RobustScaler	**23**	**76.7%**
ControlNormalizer ∩ StandardScaler	12	40.0%
StandardScaler ∩ RobustScaler	9	30.0%
All three methods	8	26.7%

Features:

ControlNormalizer

[‘103.07641’, ‘105.93981’, ‘107.9565’, ‘109.07056’, ‘109.09555’, ‘118.0709’, ‘119.07616’, ‘119.95525’, ‘149.10438’, ‘159.0712’, ‘181.01353’, ‘235.21074’, ‘45.99156’, ‘55.03962’, ‘58.94678’, ‘71.05515’, ‘71.08162’, ‘73.06527’, ‘77.05868’, ‘79.05395’, ‘83.08564’, ‘85.0697’, ‘85.09638’, ‘95.05365’, ‘95.082’, ‘96.05719’, ‘97.09225’, ‘97.10352’, ‘Age’, ‘BMI’]

StandardScaler

[‘118.0709’, ‘119.07616’, ‘119.95525’, ‘203.94814’, ‘204.9509’, ‘329.83984’, ‘330.84532’, ‘331.8501’, ‘51.03858’, ‘53.03764’, ‘58.94678’, ‘58.95657’, ‘63.02014’, ‘79.05395’, ‘85.0697’, ‘85.09638’, ‘86.10319’, ‘87.05691’, ‘87.07784’, ‘95.05365’, ‘95.082’, ‘96.05719’, ‘99.04708’, ‘99.06579’, ‘99.08365’, ‘Age’, ‘BMI’, ‘Gender’, ‘Smoking status’]

RobustScaler

[‘107.9565’, ‘108.96676’, ‘109.07056’, ‘109.09555’, ‘113.13138’, ‘118.0709’, ‘119.07616’, ‘144.91809’, ‘149.10438’, ‘159.0712’, ‘171.17475’, ‘181.01353’, ‘235.21074’, ‘45.99156’, ‘49.00474’, ‘55.03962’, ‘63.02014’, ‘64.02823’, ‘71.05515’, ‘71.08162’, ‘73.06527’, ‘79.05395’, ‘83.08564’, ‘95.05365’, ‘95.082’, ‘96.05719’, ‘97.09225’, ‘97.10352’, ‘Age’, ‘BMI’].

Conclusions.

High concordance between ControlNormalizer and RobustScaler (Full Data). The correlation of selection probabilities between ControlNormalizer and RobustScaler applied to the full dataset was 0.869, while the overlap of the top 25% feature sets comprised 23 features (76.7%). This indicates that the primary determinant of selection stability is the use of robust statistics (median and interquartile range), rather than the strategy for estimating normalization parameters (controls-only vs. full dataset). ControlNormalizer inherits the outlier robustness of RobustScaler, which is critical for PTR-TOF-MS data, where individual samples may exhibit extreme intensity values.

Low concordance with StandardScaler. The correlation between ControlNormalizer and StandardScaler was only 0.202, with an overlap of 12 features (40.0%) in the top 25%. This suggests that the sensitivity of StandardScaler to outliers and extreme values results in a fundamentally different ranking of features by stability. Under conditions of class imbalance and the presence of outliers in disease groups, StandardScaler may effectively “encode” pathological shifts into the scaling parameters, thereby reducing the reproducibility of feature selection.

Core set of stable features. Eight features were consistently selected within the top 25% by all three methods, irrespective of the normalization approach: 95.05365, 95.082, 96.05719, 97.09225, 118.0709, 119.95525, Age, and BMI. These features demonstrate maximal robustness to preprocessing variability and may be considered the most reliable candidates for further validation.

Covariates. The clinical covariates Age and BMI were included in the top 25% for ControlNormalizer and RobustScaler, but not for StandardScaler. Conversely, Gender and Smoking status appeared in the top 25% only for StandardScaler. This may reflect differences in how normalization methods handle binary and categorical variables after encoding.
